# Wired to Regulate: Brain Connectivity Predicts Emotion Regulation Capacity and Tendency

**DOI:** 10.1002/hbm.70400

**Published:** 2025-11-08

**Authors:** C. Morawetz, M. Hajrić, R. A. Rammensee, S. Berboth, U. Basten

**Affiliations:** ^1^ Department of Psychology University of Innsbruck Innsbruck Austria; ^2^ Department of Psychology RPTU University Kaiserslautern‐Landau Kaiserslautern and Landau in der Pfalz Germany; ^3^ Department of Psychiatry and Neurosciences Charité Universitätsmedizin Berlin Germany

**Keywords:** cognitive control, distraction, effective connectivity, emotion regulation, fMRI, reappraisal, resting‐state connectivity, spDCM

## Abstract

Emotion regulation relies on the flexible coordination of neural networks involved in strategy selection and implementation. While previous studies have focused on task‐related brain activity, the role of intrinsic, resting‐state connectivity in shaping regulatory tendency in strategy selection and capacity in strategy implementation remains less well understood. Using spectral Dynamic Causal Modeling (spDCM) of resting‐state fMRI data, we examined how effective connectivity within four emotion‐related brain networks predicts individual differences in the capacity to implement and the tendency to select reappraisal versus distraction for high‐intensity emotional stimuli. Forty healthy adults completed two emotion regulation tasks and a 10‐min resting‐state fMRI scan. We found that distinct and partially overlapping network dynamics predicted both strategy‐specific regulation capacity and reappraisal tendency. Notably, the fronto‐parietal and parieto‐limbic networks were central to both capacity and tendency. In addition, fronto‐lateral and limbic networks significantly contributed to the prediction of strategy‐specific measures: Reappraisal capacity was associated with broader and more inhibitory connectivity, whereas distraction capacity was related to more localized and mixed excitatory/inhibitory connectivity patterns. Crucially, the connections most predictive of distraction and reappraisal capacity were distinct rather than shared, underscoring the importance of strategy‐specific neural adaptations. These findings suggest that intrinsic brain network configurations influence the individual capacity to implement specific strategies and the tendency to select one strategy over the other.

## Graphical abstract

Resting‐state effective connectivity within emotion‐related brain networks predicts individual differences in both emotion regulation capacity and tendency, revealing distinct and overlapping neural pathways for reappraisal and distraction. These findings highlight resting‐state connectivity as a potential biomarker of regulatory traits.

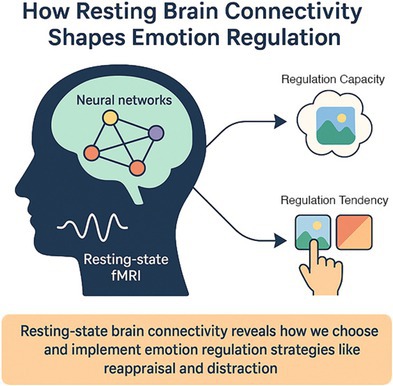

## 1 Introduction

During periods of rest, the brain remains dynamically active, engaging in complex networks of neural communication that influence a wide range of cognitive and emotional functions (Fox et al. 2005; Fox and Raichle 2007; Raichle 2015; Smith et al. 2009). Previous studies have shown that resting‐state functional magnetic resonance imaging (rs‐fMRI) connectivity, which describes these spontaneous neural interactions, provides insights into how the brain prepares for future behaviors (Chauvin et al. 2019) that influence both situational responses and overall well‐being (Sato et al. 2019). A critical determinant of emotional well‐being is emotion regulation (ER; Newman and Nezlek 2022; Quoidbach et al. 2010; Rammensee et al. 2023), a multi‐component process involving large‐scale neural networks (Morawetz, Bode, Derntl, and Heekeren 2017; Morawetz et al. 2020). This adaptive and dynamic process unfolds across several stages (Gross 2015; Sheppes 2020): identifying the need to regulate an emotion, selecting an appropriate regulation strategy, such as distraction or reappraisal, implementing the chosen strategy, and monitoring its effectiveness to adjust as necessary. By investigating how intrinsic brain networks enable the transition from emotional readiness to strategic regulation, this study aims to shed light on the neural mechanisms that underpin emotional stability and flexibility (Aldao et al. 2015; Bonanno and Burton 2013), emphasizing their contribution to the processes that govern effective ER in varying contexts.

Individual differences in the tendency to select an ER strategy, such as distraction or reappraisal, and the capacity to implement that strategy are significant predictors of well‐being and resilience (Rammensee et al. 2023). ER capacity reflects the individual's potential to use specific strategies, whereas ER tendency describes how often these strategies are selected in laboratory or real‐life situations (Silvers and Guassi Moreira 2019). While principally, people tend to choose regulation strategies for which they personally have a higher implementation capacity (Rammensee et al. 2023), the relationship between ER capacity and ER tendency is not deterministic. To better understand how the wiring of the brain, as reflected in its intrinsic connectivity, contributes to individual differences in ER capacity and tendency, we analyzed the dynamic organization of the brain's ER network during a resting state.

Few studies have related individual differences in ER to functional and/or effective connectivity during rest, and findings are mixed. Two primary approaches have been taken to link resting‐state functional connectivity to ER: To assess individual differences in ER, some have used (a) experimental measures of ER that capture individual differences in ER capacity, while others have used (b) self‐report measures on the habitual use of regulation strategies in daily life reflecting ER tendency. With the first approach, an experimental study showed positive coupling between the amygdala and the left ventrolateral prefrontal cortex (vlPFC) and insula to be related to ER capacity (Morawetz, Kellermann, et al. 2016). In contrast, another study found a negative association between amygdala and medial PFC activity in relation to ER capacity (Uchida et al. 2015). With the second approach, studies employing self‐report measures of regulatory tendency revealed a similar pattern of mixed findings, comprising positive findings on associations with connectivity between amygdala, PFC, and insula (Picó‐Pérez et al. 2018) that could not be confirmed in other studies (Uchida et al. 2015). Additionally, a replication study failed to confirm an association between self‐reported ER tendency (habitual strategy use), experimental measures of regulation capacity, and resting‐state connectivity between the amygdala and the PFC (Dörfel et al. 2020). These discrepancies may stem from differences in functional connectivity analyses and the assessment of individual differences in ER.

Recent research has demonstrated that behavioral differences in cognitive and affective processes correlate with variations in resting‐state effective connectivity (Amaoui et al. 2023; Derntl et al. 2024; Jamieson et al. 2021; Morawetz et al. 2022, 2024; Voigt et al. 2020). These studies utilized spectral Dynamic Causal Modeling (spDCM) of rs‐fMRI data to identify causal interactions among distributed brain regions (Friston et al. 2014; Park et al. 2018; Razi et al. 2015, 2017), which have been shown to predict individual differences in behavior. A recent study observed shared and distinct effective connectivity patterns for processing emotional stimuli of different intensities (Morawetz et al. 2024). For reappraisal success of low‐intensity stimuli, connectivity from frontal to temporal regions was prominent, indicating specific preparatory states. In contrast, reappraisal success for high‐intensity stimuli involved additional connections within the vlPFC and from temporal to frontal areas. Overall, rs‐fMRI effective connectivity more strongly predicted reappraisal success for low‐intensity stimuli, suggesting that connections established in the brain through regular use may facilitate ER. Conversely, high‐intensity stimuli demanded more flexible and adaptive network recruitment beyond what resting‐state connectivity alone could capture. These findings highlight the role of preparatory brain states associated with reappraisal success. However, currently, we lack an understanding of the intrinsic changes in effective connectivity that might predict the individual tendency to choose one strategy over another (such as reappraisal vs. distraction).

To address this issue, the present study examined whether individual differences in the capacity to implement distraction and reappraisal and the tendency to choose reappraisal versus distraction are associated with intrinsic brain network dynamics at rest. Building on behavioral findings linking ER capacity and ER tendency specifically for high‐intensity negative emotional stimuli (Rammensee et al. 2023), we analyzed resting‐state fMRI data using spDCM (Friston et al. 2014) to predict individual ER capacity (i.e., the capacity to implement reappraisal and distraction successfully) and tendency (i.e., the tendency to choose reappraisal over distraction) for high‐intensity emotional stimuli as assessed in two behavioral experiments. Four predefined brain networks central to emotion generation and regulation, identified in a previous meta‐analysis (Morawetz et al. 2020), served as a framework for our analyses: network 1 (fronto‐parietal, response inhibition/executive control), network 2 (fronto‐temporal, appraisal/language processing), network 3 (temporo‐limbic, emotion generation), and network 4 (parieto‐limbic, physiological responses). Our analysis focused on common and distinct associations of neural connectivity patterns with ER capacity and tendency to assess how effective connectivity within these networks predicts behavioral outcomes. It targeted, *directed*, theory‐motivated couplings within ER‐relevant networks to maximize mechanistic interpretability—that is, identifying which pathways relate to regulation capacity and selection tendency. While multivariate models that aggregate across connections may improve prediction, they can obscure the contributing mechanisms and, without external validation, risk optimistic bias. We therefore focus on edge‐level inferences.

We hypothesized that effective connectivity within the ER networks during rest would predict reappraisal capacity measured as individual regulation success (c.f. Morawetz et al. 2024). Given the exploratory nature of this study, we anticipated distinct connectivity patterns to be associated with distraction/reappraisal capacity and tendency, but did not formulate specific directed hypotheses. Generally, we expected network 1 and network 2—networks primarily involved in ER—to show stronger predictive relations with ER capacity and tendency than networks 3 and 4, a hypothesis tested using Leave‐One‐Out Cross‐Validation (LOOCV). Here, our goal was to test whether intrinsic, trait‐like network dynamics at rest scaffold individual differences in emotion regulation capacity (implementation) and tendency (selection). Accordingly, we interpret resting‐state effective connectivity as a baseline architectural feature rather than as a direct proxy for regulation processes during task performance. To this end, we used resting‐state fMRI with spDCM to quantify directed coupling within ER‐relevant networks. We did not acquire fMRI during the ER tasks in this dataset; thus, our claims pertain to trait‐level preparatory architecture rather than state‐evoked responses during regulation.

## 2 Materials and Methods

### 2.1 Participants

Forty right‐handed, healthy participants with normal or corrected to normal vision (20 females, mean age = 22.53 years, SD = ±3.76, range = 18–35) participated in the behavioral and the fMRI study. All participants gave written informed consent and reported no history of neurological or psychiatric disorders. Participants were compensated with money, and no participant had to be excluded due to excessive movement (> 3 mm) during the rs‐fMRI scanning. The study was approved by the local ethics committee of the Goethe University of Frankfurt, Germany (#2016‐10) and carried out under the Declaration of Helsinki.

### 2.2 Experimental Design

#### 2.2.1 Procedure

Participants completed the study across multiple sessions. Owing to COVID‐19–related scheduling constraints, the interval between the rs‐fMRI session (session 1) and the behavioral testing varied across participants (M = 534 days; SD = ±101; range = 420–672 days). Because rs‐fMRI is commonly interpreted as a trait‐like measure of intrinsic network architecture, we expected these metrics to be relatively stable over longer time intervals (Choe et al. 2015; Horien et al. 2019; Noble et al. 2019; Shehzad et al. 2009). Nevertheless, we included the rs‐fMRI‐to‐behavioral interval (mean z‐transformed timelag = −3.83 days; SD = ±0.98) as a control variable in all analyses.

Session 1 (MRI #1; ~60 min): Inside the scanner, participants first performed a value‐based decision‐making task (~50 min; Basten et al. 2010), followed by a 10‐min resting‐state fMRI (rs‐fMRI) run. During rs‐fMRI, participants were instructed to keep their eyes open, fixate on a central cross, remain still, and stay awake. Between sessions, from home, participants completed online questionnaires assessing personality and mental health. Session 2 (MRI #2; task‐fMRI): Participants returned for a second MRI visit that included a reward sensitivity task and an interpretation bias task (not analyzed for the present study). Behavioral laboratory session (separate visit): Participants were tested individually at a desktop computer and completed, in fixed order, the ER *implementation* task followed by the ER *selection* task (see Rammensee et al. 2023). During the ER implementation task, participants familiarized themselves with the regulation strategies of reappraisal and distraction, which they could subsequently choose between in the ER selection task. After completion of both ER tasks, they rated the arousal elicited by each aversive image on a 1–9 scale (Bradley and Lang 1994), providing individual stimulus‐intensity indices.

#### 2.2.2 Stimuli

As stimuli in the ER tasks, we used aversive images from the International Affective Picture System (IAPS; Bradley and Lang 2007). Of note, while stimulus selection was guided by normative valence and arousal ratings to balance content and stimulus intensity, the intensity variable used for the here‐reported analysis was defined at the individual level: images were categorized into low and high intensity based on a within‐participant median split of each participant's own arousal ratings (1–9 scale) collected after the ER tasks (see Section 2.2.5). The ER implementation task included *n* = 60 pictures (mean arousal = 5.53, SD = ±2.20, mean valence = 2.69, SD = ±1.53; according to normative ratings from Bradley and Lang 2007). The ER selection task included *n* = 30 pictures (mean arousal = 5.56, SD = ±2.13; mean valence = 2.70, SD = ±1.57). Both tasks included an equal number of high‐ and low‐intensity pictures. Previous research established a positive correlation between ER capacity and ER tendency only for high‐intensity stimuli, that is, the better participants were at decreasing their negative affect using reappraisal in contrast to distraction, the more they tended to select reappraisal in response to high‐intensity stimuli (Rammensee et al. 2023). Therefore, the current study focused on the ER capacity and ER tendency for high‐intensity stimuli and their relation to changes in effective connectivity. The high‐intensity stimuli in the ER implementation task showed a mean valence of 1.91 (SD = ±0.30) and arousal of 6.25 (SD = ±0.62). The high‐intensity stimuli in the ER selection task had a mean valence of 1.99 (SD = ±0.32) and arousal of 6.12 (SD = ±0.69).

#### 2.2.3 ER Implementation Task

**FIGURE 1:**
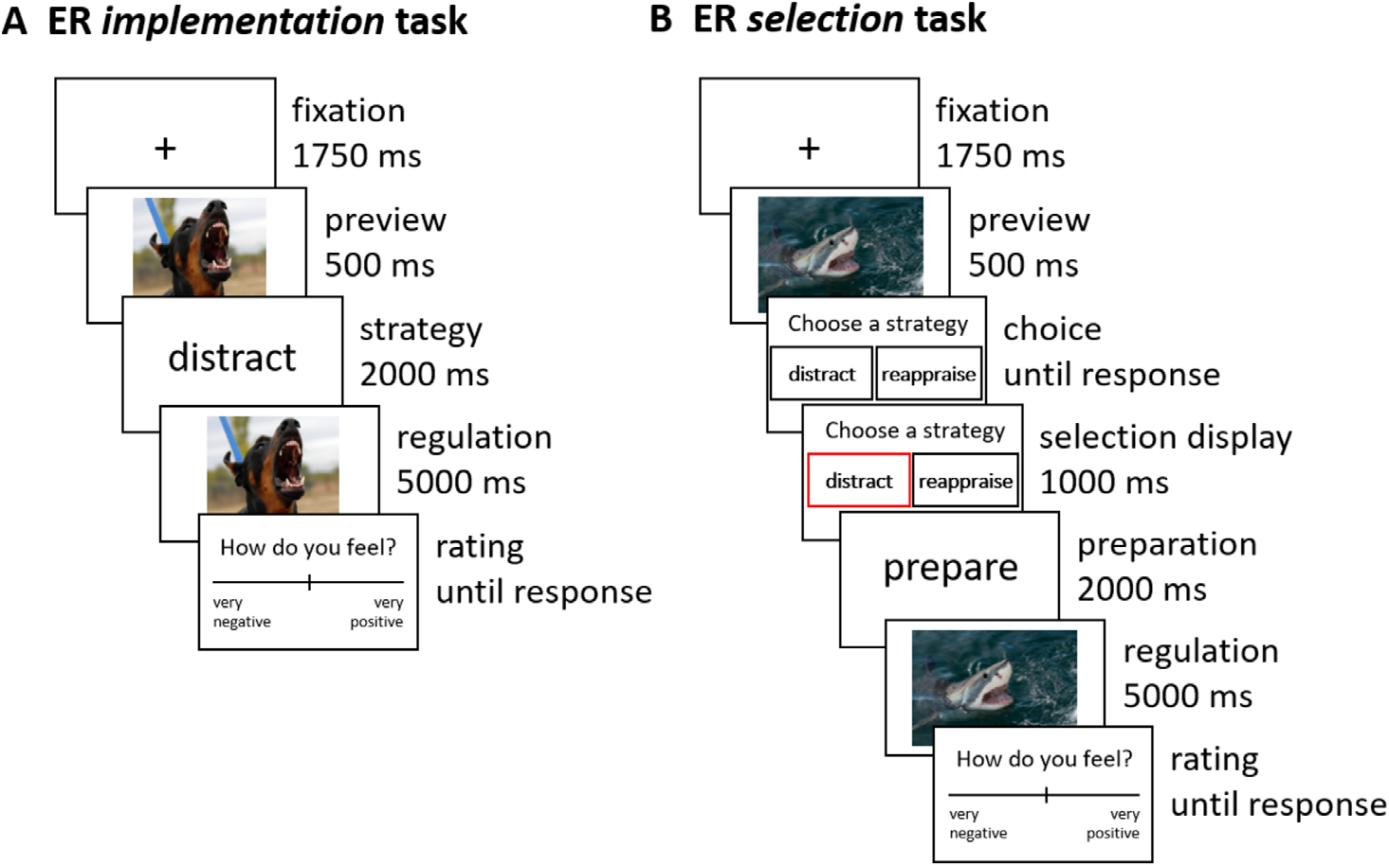
Emotion regulation tasks. Schematic overview of trial structure. (A) Emotion regulation implementation task. (B) Emotion regulation selection task. Trial sequences are illustrated with freely available images that were not used in the current study.

The ER implementation task assessed the individual capacity to implement reappraisal and distraction in the face of high‐intensity stimuli (Rammensee et al. 2023) (Figure 1A). Before the ER implementation task, participants received detailed instructions on the regulation strategies, did practice trials, and received feedback from the experimenter. The main task included 60 trials (30 low‐ and 30 high‐intensity negative images). Participants first saw a 500 ms preview of the image, followed by a 2 s strategy instruction, which was randomized between distraction (distract), reappraisal (reinterpret), and a no‐regulation control condition (look). For distraction, participants were told to think of something neutral and unrelated to the image. For reappraisal, participants were asked to reinterpret the image's meaning more positively or less negatively. Participants were instructed to look at the images without regulating their emotions in the control condition. After seeing the strategy instruction, participants were presented with the aversive image for 5 s and subsequently asked to rate their affective state at the end of the trial on a scale from −200 (very negative) to +200 (very positive). For an overview of trial timing, see Figure 1A.

#### 2.2.4 ER Selection Task

The ER selection task assessed participants' tendency to select reappraisal versus distraction (Sheppes et al. 2011). In contrast to the implementation task, in the selection task, participants could freely choose which strategy they preferred to implement. The trial structure of the ER selection task was similar to the implementation task, with one significant difference. Participants had to choose between distraction and reappraisal after seeing the image preview in the selection task. After the participants indicated their decision, a red frame highlighted the selected strategy, and (as in the ER implementation task), participants had 2 s to prepare for its implementation. This task included 30 trials (15 low‐ and 15 high‐intensity images). During the 5 s presentation of the picture, participants implemented the selected regulation strategy. Subsequently, as in the ER implementation task, they rated their affective state on a scale from “very negative” to “very positive”. For an overview of trial timing, see Figure 1B.

#### 2.2.5 Individual Ratings of Stimulus Intensity

After completing the ER tasks, participants rated the arousal elicited by each aversive image on a 1–9 Likert scale using the Self‐Assessment Manikin scale (Bradley and Lang 1994). For analyses, we computed each participant's median arousal across all rated images and classified trials as low intensity (≤ median) or high intensity (> median). Intensity was thus based solely on individual arousal, not normative valence or arousal; normative ratings were used only for initial stimulus selection.

### 2.3 Behavioral Data Analysis

Individual ER capacity was calculated using data from the ER implementation task. It was quantified as the difference between the trial‐wise individual affect rating in the regulation conditions (distraction or reappraisal) minus the mean across all trials from the control condition (look). If this difference was > 0, the trial was considered successful. The total number of successful trials was divided by the total number of trials overall to get the percentage of successful trials as established in similar designs (Morawetz et al. 2024). This resulted in two measures of regulation capacity: distraction capacity and reappraisal capacity. Individual reappraisal tendency (i.e., the tendency to choose reappraisal over distraction) was calculated using data from the ER selection task. Individual reappraisal tendency was computed as the mean of all choices where reappraisal was coded as 1 and distraction as −1. This resulted in a variable with scores ranging from −1 (100% distraction) over 0 (50% distraction and 50% reappraisal) to 1 (100% reappraisal). A reappraisal tendency score of 0.75 indicates that the participant chose reappraisal in approximately 87.5% of all trials. As a previous study found individual differences in ER capacity and tendency to be related to high‐intensity stimuli specifically (Rammensee et al. 2023), we focused our analyses on the (individually defined) high‐intensity trials.

### 2.4 Behavior Reliability Analysis

We estimated internal consistency for our key ER behavioral indices—distraction capacity, reappraisal capacity, and reappraisal tendency (free‐choice proportion of reappraisal vs. distraction)—using a permutation‐based split‐half approach. Specifically, we generated 5000 random splits of trials per participant and computed split‐half correlations for each index using the *splithalf* package (version 0.7.2; Parsons 2020) with custom wrapper code. Reliabilities were Spearman–Brown corrected, and 95% confidence intervals were derived from the empirical distribution across permutations. This approach provides a robust estimate of internal consistency that is not tied to a particular split (e.g., odd–even vs. first–second half).

### 2.5 RS‐FMRI Data Acquisition

Resting‐state scans lasted 10 min. Participants were instructed to keep their eyes open, fixate on a central cross, remain still, and stay awake. Whole‐brain functional and anatomical images were acquired using a SIEMENS Magnetom Trio 3.0 Tesla MR scanner with an 8‐channel head coil. A high‐resolution three‐dimensional (3D) T1‐weighted dataset was acquired for each participant (MP‐RAGE sequence: 1 mm isometric voxel size, 192 sagittal slices, FoV 256 × 256 × 192 mm^3^, TR 1.9 s, TE 2.6 ms). For rs‐fMRI, an EPI sequence was used (TR = 2.3 s; TE = 30 ms; 42 slices; voxel size = 3.0 × 3.0 × 2.7 mm^3^; 2.7 mm slice thickness; field of view = 192 × 192 × 169 mm^3^; GRAPPA parallel imaging acceleration factor 2 [32 reference lines], flip angle = 70°; number of rs‐fMRI volumes: 260; negative phase blip polarity; z‐shim amplitude −1.5 mT/m).

### 2.6 Neuroimaging Analysis

**FIGURE 2:**
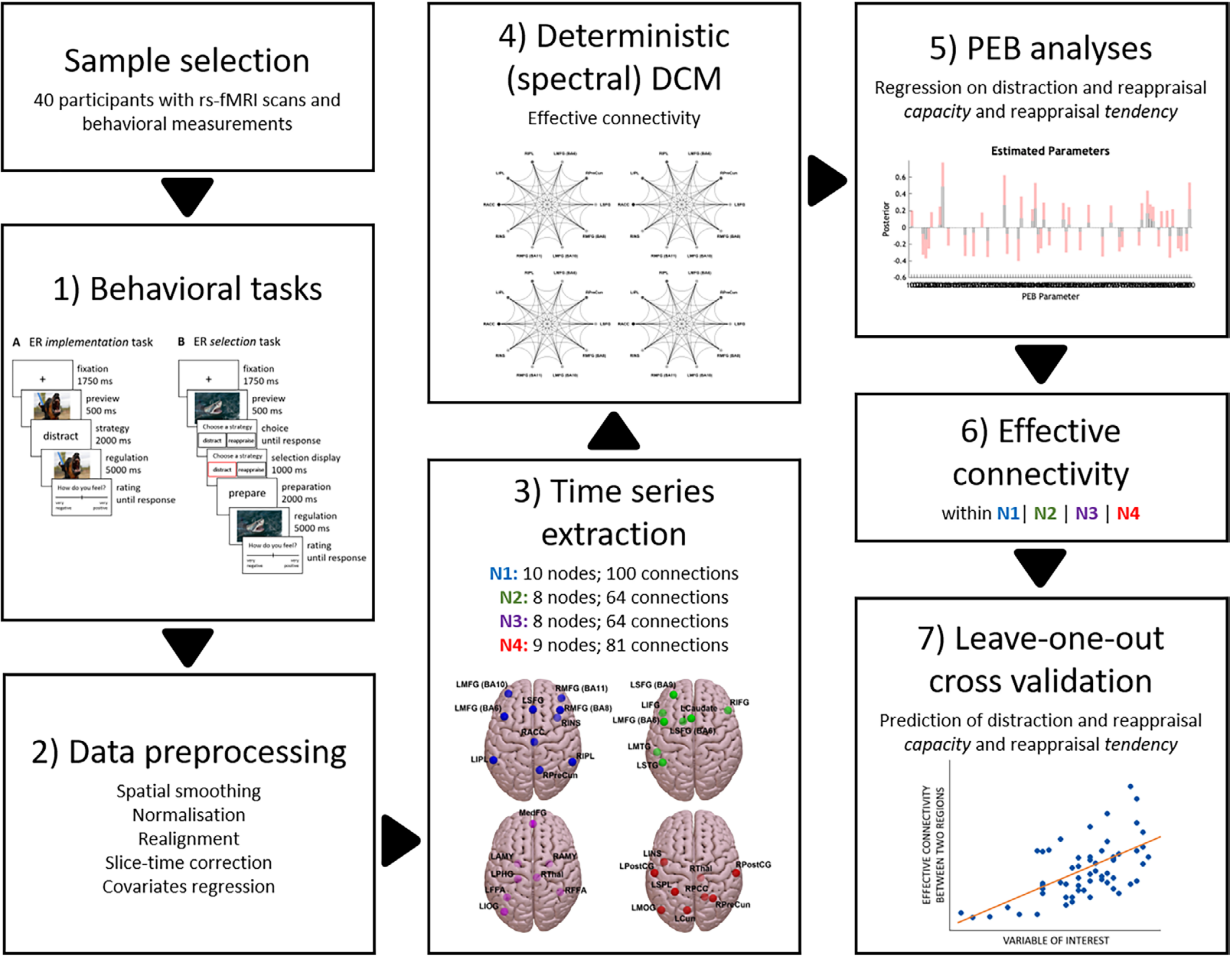
RS‐FMRI data analysis pipeline. (1) Computation of the behavioral variables from the ER implementation and selection tasks. (2) Preprocessing of rs‐fMRI data. (3) Time series extraction from the four predefined networks (N1–N4). For interpretive context, functional associations derived from BrainMap are reported in our original paper (Morawetz et al. 2020); here, we refrain from function‐based names because these networks are not specific to a single cognitive or affective processes. (4) Spectral dynamic causal modelling analysis with fully connected models. (5) PEB = Parametric Empirical Bayes analyses for regression of effective connectivity on ER capacity and tendency. (6) Identification of the modulation effect of ER capacity and ER tendency on effective connectivity. (7) Leave‐one‐out cross‐validation to predict ER capacity and tendency.

An overview of the analytical pipeline is given in Figure 2.

#### 2.6.1 STEP 1: Image Preprocessing

Rs‐fMRI data were analyzed using SPM12 (Statistical Parametric Mapping, Wellcome Centre for Human Neuroimaging, London, UK) in Matlab R2021a (MathWorks, Natick, MA). The standard preprocessing steps included slice‐time correction, realignment to the mean image, coregistration to the respective structural image of the participant, spatial normalization to the standard EPI template (Montreal Neurological Institute, MNI), and smoothing with an isotropic Gaussian kernel (6 mm full‐width at half‐maximum). Functional images were realigned to the first volume to estimate six rigid‐body motion parameters (three translations, three rotations). Nuisance regression removed six motion parameters and mean white‐matter and cerebrospinal fluid signals from each ROI time series. Time series were inspected for visible artifacts; participants exceeding 3 mm translation or 3° rotation would have been excluded (none did).

#### 2.6.2 STEP 2: Region‐Of‐Interest Selection and Time Series Extraction

Four networks were taken from a previous meta‐analysis on ER. Each network (N1–N4) consists of several regions of interest (ROIs) (Morawetz et al. 2020). These ROIs are implicated in emotion generation and regulation within their respective neural networks. Network labels are kept function‐neutral to avoid reification of single‐function interpretations. Functional associations for these networks were characterized via BrainMap decoding in our original report (Morawetz et al. 2020), and we refer readers there for details. Here, we provide anatomical descriptors for orientation. N1 (fronto‐parietal) is involved in attention, working memory, and executive control, and consists of frontal and parietal ROIs (e.g., medial frontal gyri and inferior parietal lobules). N2 (fronto‐temporal) is primarily a language network, consisting of frontal and temporal ROIs (e.g., inferior frontal gyri and temporal gyri). N3 (temporo‐limbic) is a subcortical network comprising limbic structures such as the amygdalae, which are key to emotion processing (Thompson and Neugebauer 2019; Wager et al. 2008). N4 (parieto‐limbic) consists of regions such as the postcentral gyri and insula, serving as a hub between the first three networks. For an overview of the MNI coordinates of all ROIs, refer to Figure S1 and Table S1. After preprocessing of the data, the BOLD signal intensities over time were extracted for all ROIs and all participants, resulting in a temporal series of neural activity for each region. ROI time series were extracted using predefined spherical masks centered on the MNI coordinates listed in Table S1. Each mask was a 6‐mm radius sphere. For each ROI, we computed the mean BOLD time series across all voxels within the sphere after nuisance regression (6 motion parameters, WM, CSF).

#### 2.6.3 STEP 3: Spectral Dynamic Causal Modeling

SPM12 and DCM12 were used to perform the spDCM analysis and estimate the effective connectivity between the predefined ROIs in all four networks. First, four fully connected models (N1–N4) for all participants were created to examine specific connections between the ROIs within each network (Figure S2). To estimate the effective connectivity parameters of the fully connected models based on the observed cross‐spectral density, these models were inverted using spectral DCM (spDCM; Razi et al. 2015). The strength, direction, and modulatory effects of the connections between brain regions can be observed by estimating the parameters within the fully connected models (i.e., between source and target brain regions). These estimated parameters allow for quantifying the influence and impact of each connection in Hertz (Hz) and provide information on the effective connectivity within the brain networks. Post hoc model convergence statistics were checked for variance explained, the largest absolute parameter estimates, and the effective number of parameters estimated for all final reduced models.

The average percentage of explained variance of all participants' final models was very high (N1: mean = 89.09, SD = ±19.98; N2: mean = 90.29, SD = ±2.19; N3: mean = 88.93, SD = ±4.51; N4: mean = 89.2, SD = ±2.45; Figure S3), indicating excellent model convergence. In addition, low posterior correlations among all parameters were observed within all networks (Figure S4). This means each parameter provided unique information and was not highly dependent on other parameters.

After fully inverting the models for all participants, Bayesian model reduction (Friston 2011) was employed to find the models that best explained the data. This model reduction computes the posterior probability for the parameters and the model evidence for any nested model within the full models. After the reduction, Bayesian model averaging estimates the models weighted by their model evidence and thresholds the parameters. A conservative threshold of posterior probability > 0.99 was used only to include parameters with strong evidence of an effect (Kass and Raftery 1995).

**FIGURE 3:**
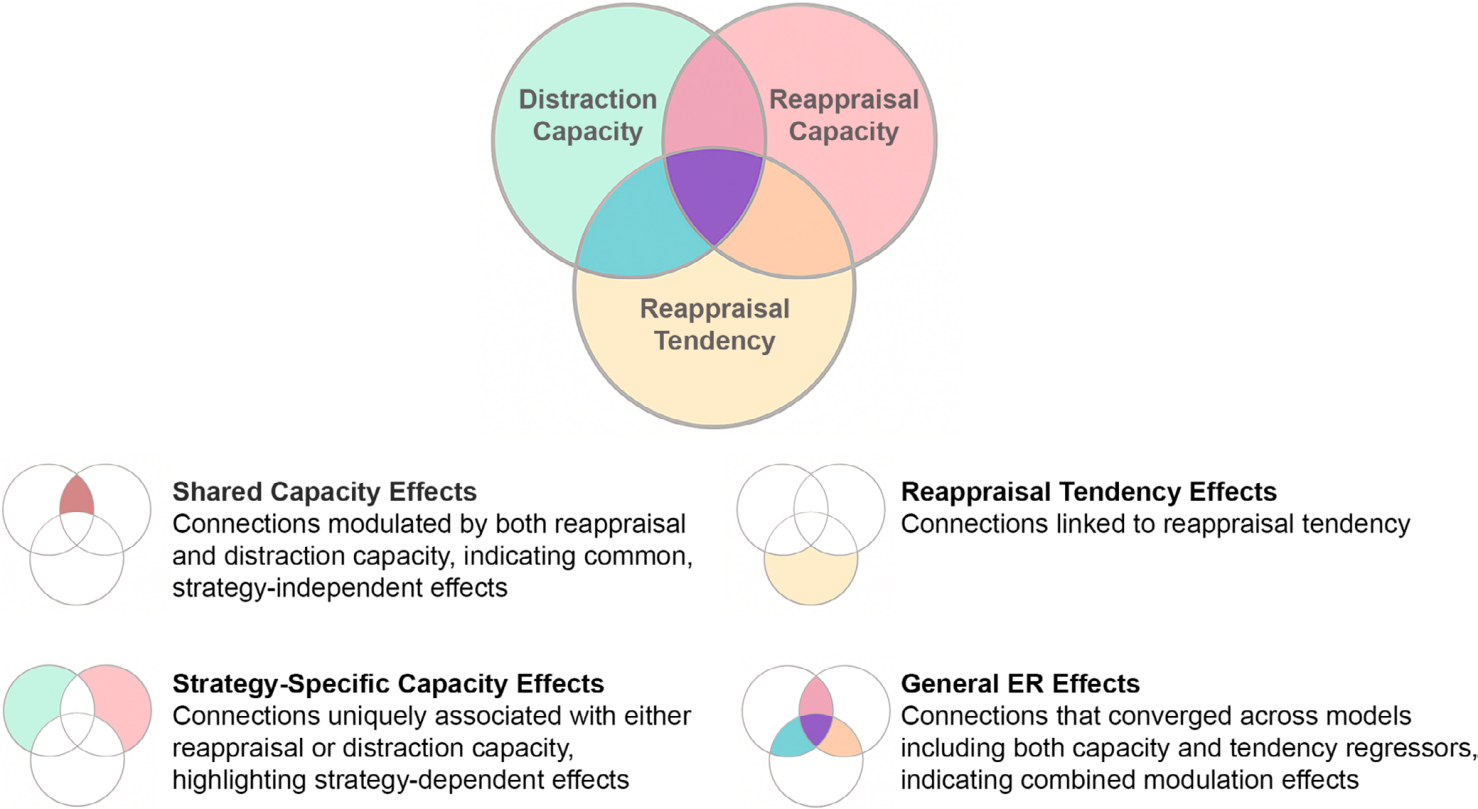
Analysis steps for the spectral dynamic causal modeling analysis. Analysis steps for estimating shared capacity effects, strategy‐specific capacity effects, reappraisal tendency effects, and general ER effects.

The hierarchical Parametric Empirical Bayes (PEB) framework for DCM (Friston et al. 2016) was used as a second‐level analysis to examine how effective connectivity within networks N1–N4 was modulated by distraction and reappraisal capacity as well as by reappraisal tendency during high‐intensity trials. Separate PEB models were estimated for each network and each ER measure, resulting in eight models for capacity (*reappraisal_capacity*_*N1‐N4*_, *distraction_capacity*_*N1‐N4*_) and four models for reappraisal tendency (*reappraisal_tendency*_*N1‐N4*_). In each PEB model, reappraisal capacity, distraction capacity, or reappraisal tendency served as the regressor of interest, while age, sex, and timelag were included as regressors of no interest. Thus, each model contained one ER measure of interest (e.g., *reappraisal_capacity*_*N1*_) and three covariates of no interest (*age, sex*, and *timelag*). To allow for interpretable intercepts representing average connectivity, all ER measures were mean‐centered. All steps of the spDCM and PEB pipeline are illustrated in Figure 3.

To guide interpretation, in the presentation of results, we grouped the model outcomes into four conceptual categories: (1) connections modulated by both ER capacity measures, potentially reflecting a shared, strategy‐independent ER capacity (*shared capacity effects*); (2) connections uniquely associated with either reappraisal or distraction capacity (*strategy‐specific capacity effects*); (3) connections linked to reappraisal tendency (*reappraisal tendency effects*); and (4) connections identified across models for both capacity and tendency measures (*general ER effects*). These categories do not represent separate statistical analyses, but provide a frame for the comparative summary of model outcomes across PEB models.

#### 2.6.4 STEP 4: Leave‐One‐Out Cross‐Validation

In the last step, a leave‐one‐out cross‐validation (LOOCV) was used to examine the predictive validity of the obtained connections. This part of the analysis assessed whether the effect size of the observed associations allowed for the prediction of a left‐out participant's ER capacity or ER tendency from the connectivity within the networks N1–N4 for each significant connection separately. This was achieved by iteratively training the model on all observations but one, leaving out one observation at a time (Zeidman et al. 2019). Only significant effect sizes that were large enough to predict ER capacity or ER tendency with an out‐of‐sample estimate are reported (*p* < 0.05).

## 3 Results

### 3.1 Behavioral Data

**FIGURE 4:**
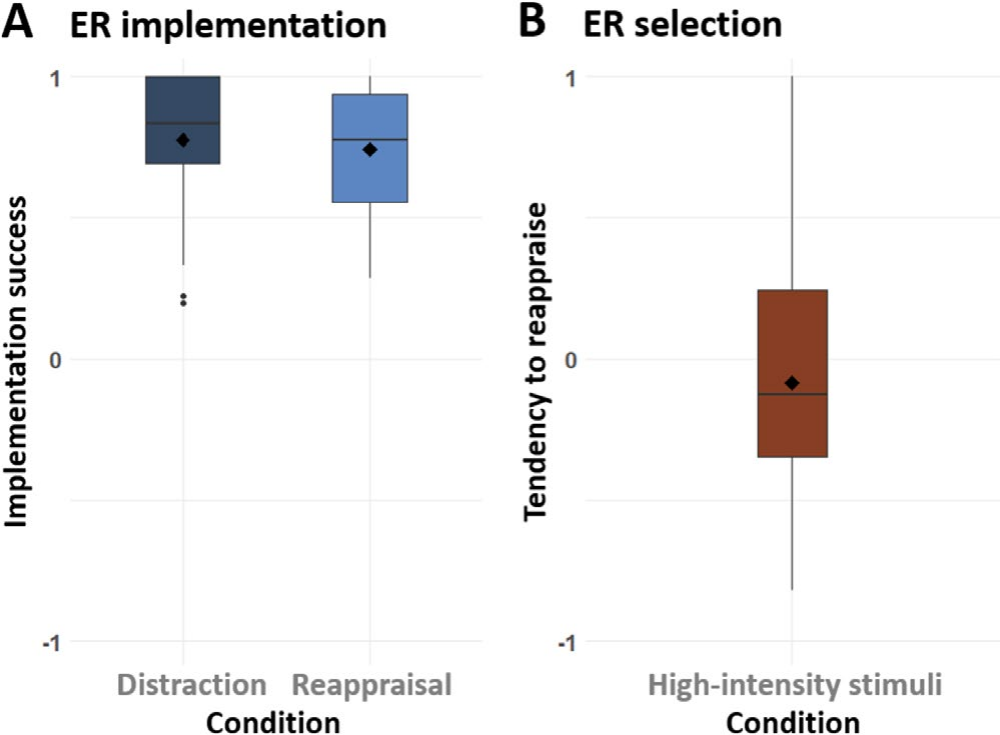
Mean implementation success and regulation tendency in the ER tasks. (A) ER implementation task. Mean ER implementation success for distraction and reappraisal of high‐intensity images. *Y*‐axis denotes the percentages of the successful trials converted to a range from −1 to 1. (B) ER selection task. Mean tendency to reappraise for high‐intensity images. *Y*‐axis shows relational values ranging between −1 (100% distraction) and 1 (100% reappraisal).

In the ER implementation task, for both regulation strategies, ER implementation success was significantly different from zero (distraction: *t*(39) = 21.62, *p* < 0.001, *d* = 3.54, 95% CI [0.705, 0.851]; reappraisal: *t*(39) = 21.62, *p* < 0.001, *d* = 3.53, 95% CI [0.673, 0.812]) (Figure 4A). There was no significant difference in ER success between distraction (*M* = 0.78, SD = ±0.22) and reappraisal (*M* = 0.74, SD = ±0.21) (*t*(39) = 1.02, *p* = 0.313, *d* = 0.16, 95% CI [−0.035, 0.106]). In the ER selection task, the tendency to select reappraisal over distraction did not significantly differ from zero (*M* = −0.09, SD = ±SD = ±0.42), *t*(39) = −1.263, *p* = 0.214, *d* = −0.21, 95% CI [−0.220, 0.051] (Figure 4B).

### 3.2 Behavioral Reliability

Permutation‐based split‐half estimates indicated moderate internal consistency for the ER indices. Mean Spearman–Brown–corrected reliabilities [95% CI] were 0.50 [0.25, 0.70] for distraction capacity, 0.39 [0.05, 0.66] for reappraisal capacity, and 0.59 [0.39, 0.75] for reappraisal tendency. Notably, the tendency estimate aligns with prior work (Rammensee et al. 2023: 0.66 [0.60, 0.72]).

### 3.3 Shared ER Capacity Effects

**FIGURE 5:**
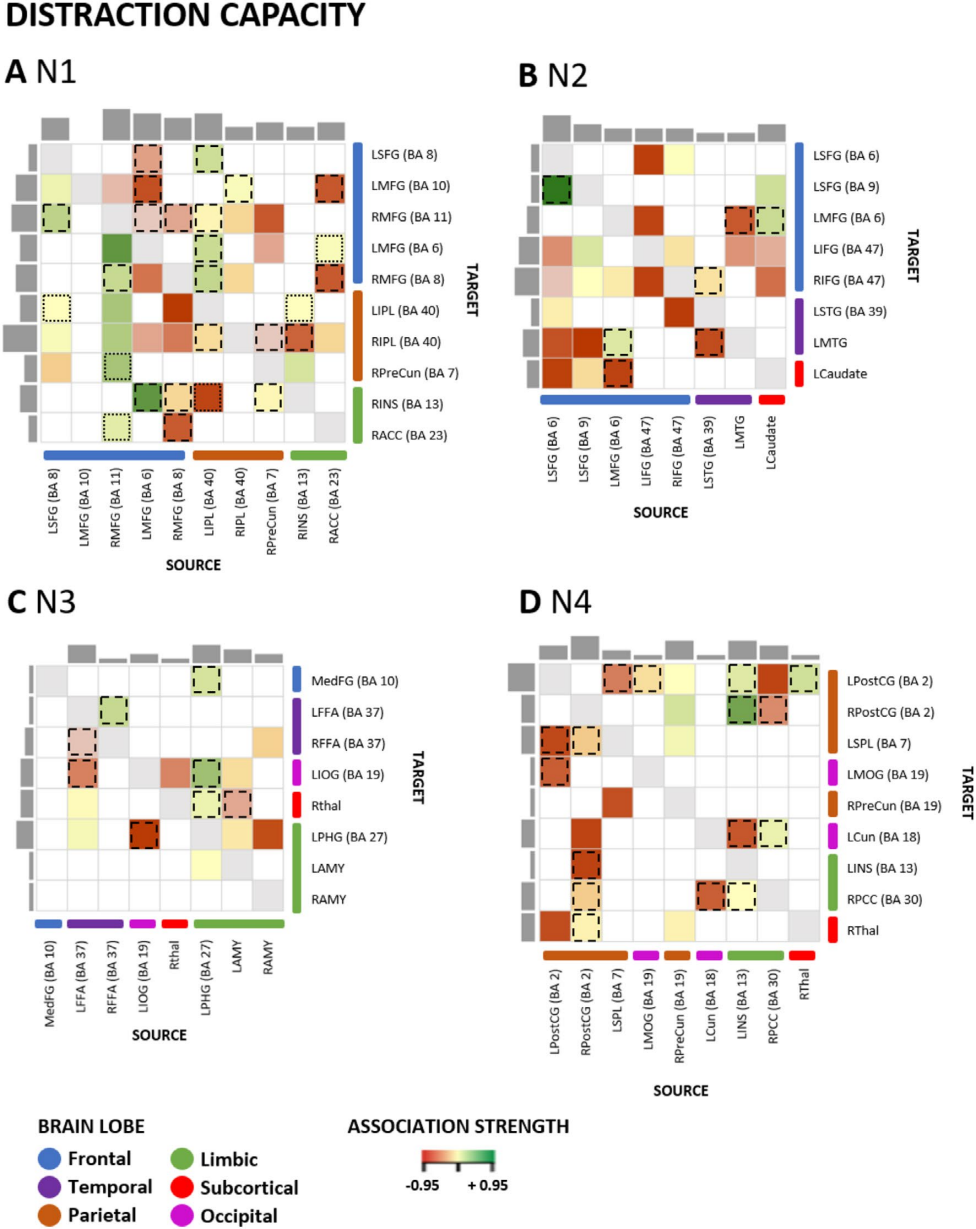
Distraction capacity and effective connectivity within ER networks N1–N4. Association between distraction capacity for high‐intensity negative stimuli and EC within each network (N1–N4). Association strength: The colors in the heat map characterize the association of the ER variable with the EC, with green indicating a positive and red a negative association measured in Hertz (Hz). Dashed lines highlight an overlap in associations for distraction and reappraisal capacity with identical directionality. Dotted lines indicate an overlap in associations for distraction and reappraisal capacity with different directionality (i.e., positively or negatively associated with EC). Only associations exceeding a conservative threshold of posterior probability > 0.99 are reported. Matrix columns: source regions; rows: target regions. The number of outputs and inputs of specific regions is illustrated with the gray bars on the side (inputs) and top (outputs) of the matrix. BA, Brodmann area; LAMY, left amygdala; LCaudate, left caudate; LCun, left cuneus; LFFA, left fusiform face area; LIFG, left inferior frontal gyrus; LINS, left insula; LIOG, left inferior occipital gyrus; LIPL, left inferior parietal lobule; LMFG, left middle frontal gyrus; LMOG, left middle occipital gyrus; LMTG, left middle temporal gyrus; LPHG, left parahippocampal gyrus; LPostCG, left postcentral gyrus; LSFG, left superior frontal gyrus; LSPL, left superior parietal lobule; LSTG, left superior temporal gyrus; MedFG, medial frontal gyrus; RACC, right anterior cingulate cortex; RAMY, right amygdala; RFFA, right fusiform face area; RIFG, right inferior frontal gyrus; RINS, right insula; RIPL, right inferior parietal lobule; RMFG, right middle frontal gyrus; RPCC, right posterior cingulate; RPostCG, right postcentral gyrus; RPreCun, right precuneus; RThal, right thalamus.

**FIGURE 6:**
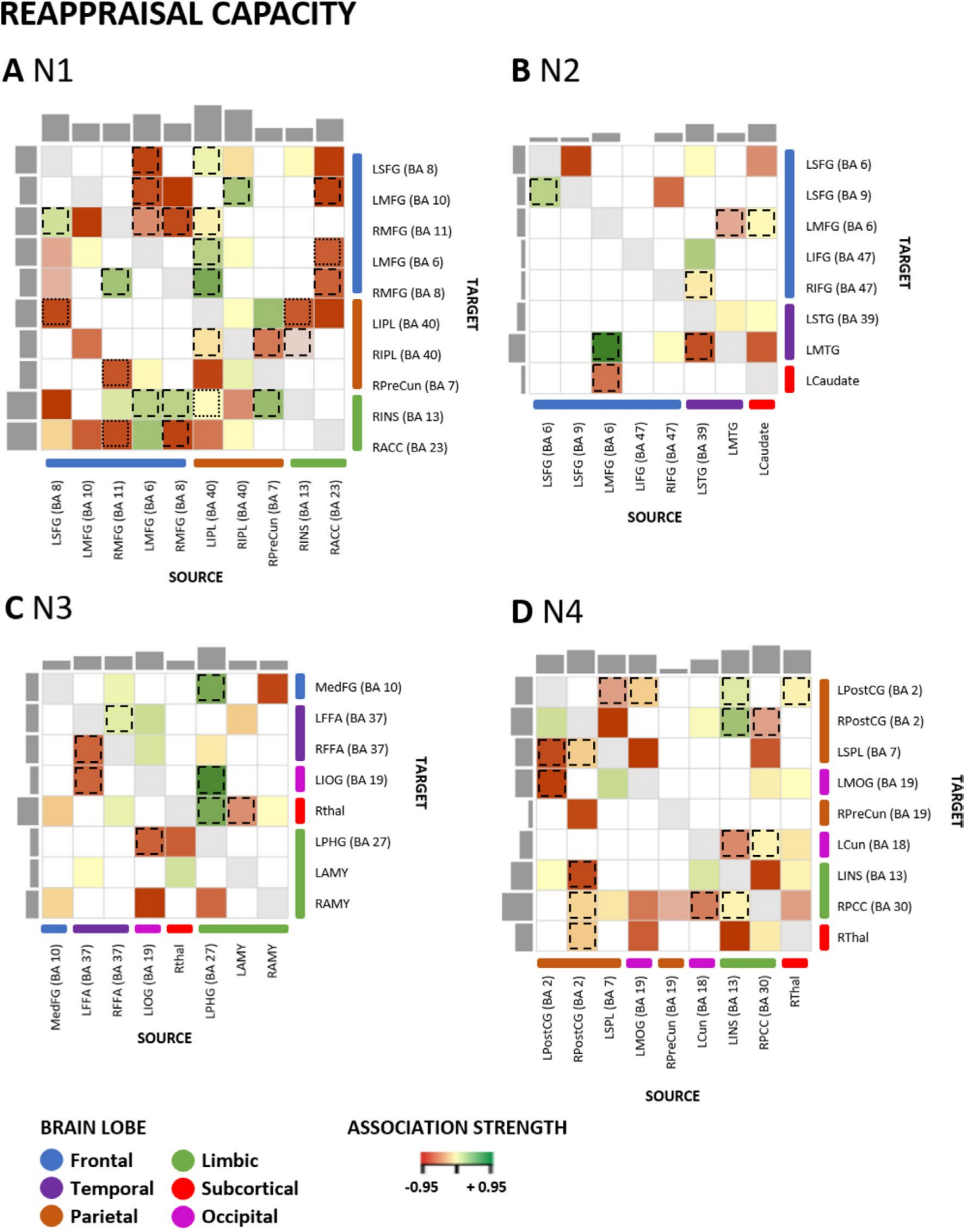
Reappraisal capacity and effective connectivity within ER networks N1–N4. Association between reappraisal capacity for high‐intensity negative stimuli and EC within each network (N1–N4). Association strength: The colors in the heat map characterize the association of the ER variable with the EC, with green indicating a positive and red a negative association measured in Hertz (Hz). Dashed lines highlight an overlap in associations for distraction and reappraisal capacity. Dotted lines indicate an overlap in associations for distraction and reappraisal capacity with different directionality (i.e., positively or negatively associated with EC). Only associations exceeding a conservative threshold of posterior probability > 0.99 are reported. The number of outputs and inputs of specific regions is illustrated with the gray bars on the side (inputs) and top (outputs) of the matrix. BA, Brodmann area; LAMY, left amygdala; LCaudate, left caudate; LCun, left cuneus; LFFA, left fusiform face area; LIFG, left inferior frontal gyrus; LINS, left insula; LIOG, left inferior occipital gyrus; LIPL, left inferior parietal lobule; LMFG, left middle frontal gyrus; LMOG, left middle occipital gyrus; LMTG, left middle temporal gyrus; LPHG, left parahippocampal gyrus; LPostCG, left postcentral gyrus; LSFG, left superior frontal gyrus; LSPL, left superior parietal lobule; LSTG, left superior temporal gyrus; MedFG, medial frontal gyrus; RACC, right anterior cingulate cortex; RAMY, right amygdala; RFFA, right fusiform face area; RIFG, right inferior frontal gyrus; RINS, right insula; RIPL, right inferior parietal lobule; RMFG, right middle frontal gyrus; RPCC, right posterior cingulate; RPostCG: right postcentral gyrus; RPreCun, right precuneus; RThal, right thalamus.

In the first PEB models, the connections generally involved in the capacity to regulate emotions were of interest (i.e., connections demonstrating an association with both reappraisal and distraction capacity). These are highlighted in Figures 5 and 6 with dashed and dotted lines. The direction of the connectivity between regions (excitatory/inhibitory) across the sample can be seen in Table 2.

For all four networks, there were substantial overlaps in the connections that were associated with distraction and reappraisal capacity. Approximately half of the connections that were associated with distraction capacity also showed an association with reappraisal capacity (Table 1). In contrast, reappraisal capacity showed more distinctive connections in N1. In addition, several connections within N1 had differing associations with distraction and reappraisal capacity (i.e., a positive association with one covariate and a negative association with the other). All overlapping connections observed in networks N2, N3, and N4 had the same direction of association with distraction and reappraisal capacity (i.e., either a positive or negative association with both).

**TABLE 1:** PEB results overview.

*General overview for distraction and reappraisal capacity, and reappraisal tendency*							
Behavioral covariate	Network	Total connections	Predictive connections	Connection type			Interconnectivity and [inhibitory/excitatory]
				Inhibitory [%] and [±cov. association]	Excitatory [%] and [±cov. association]	Highest I/O connections and ROI [number of connections]	
Distraction capacity	N1	43	23	56 [10/14]	44 [7/12]	RMFG (BA11) [13]	10 frontal [5/5], 2 parietal [1/1]
	N2	27	7	59 [11/5]	41 [4/7]	RIFG [9]	11 frontal [2/9], 1 temporal [1/0]
	N3	16	2	31 [2/3]	69 [4/7]	LPHG [8]	2 temporal [0/2], 3 limbic [2/1]
	N4	24	4	38 [6/3]	62 [5/10]	LPostCG [9]	7 parietal [3/4], 1 limbic [0/1]
Total		110	36	[29−/25+]	[20−/36+]		
Reappraisal capacity	N1	50	25	60 [14/16]	40 [12/8]	LIPL [13]	11 frontal [5/6], 6 parietal [3/3]
	N2	16	6	69 [5/6]	31 [2/3]	LSTG, LMTG [6]	3 frontal [1/2], 2 temporal [2/0]
	N3	23	9	57 [3/10]	43 [5/5]	RThal, LPHG [7]	2 temporal [0/2], 1 limbic [1/0]
	N4	37	19	59 [11/11]	41 [6/9]	RPCC [13]	6 parietal [3/3], 2 limbic [1/1]
Total		126	59	[33−/43+]	[25−/25+]		
Reappraisal tendency	N1	38	16	58 [14/8]	42 [9/7]	RPreCun, RMFG (BA11) [10]	7 frontal [4/3], 4 parietal [2/2], 1 limbic [1/0]
	N2	30	6	57 [6/11]	43 [7/6]	LMFG [9]	10 frontal [3/7]
	N3	24	4	38 [5/4]	62 [8/7]	RThal, LAMY [9]	3 limbic [1/2]
	N4	26	10	50 [5/8]	50 [10/3]	LINS [8]	3 parietal [2/1], 1 occipital [0/1], 2 limbic [2/0]
Total	118	36	[30−/31+]	[34−/23+]			

*Distinctive connections for distraction capacity and reappraisal capacity*					
Behavioral covariate	Network	Specific connections	Connection type		Specific % of total DC/RC connections
			Inhibitory [%] and [±cov. association]	Excitatory [%] and [±cov. association]	
Distraction capacity	N1	17	53 [3/6]	47 [4/4]	40
	N2	20	50 [8/2]	50 [4/6]	74
	N3	8	25 [1/1]	75 [1/5]	50
	N4	8	25 [1/1]	75 [3/3]	33
Reappraisal capacity	N1	24	50 [7/5]	50 [5/7]	48
	N2	9	78 [3/4]	22 [1/1]	56
	N3	15	67 [2/8]	33 [2/3]	65
	N4	21	71 [6/9]	29 [4/2]	57

Connections modulated by distraction capacity and reappraisal capacity					
Behavioral covariate	Network	Commonly modulated	Connection type		Overlap % of total DC/RC connections
			Inhibitory [%] and [±cov. association]	Excitatory [%] and [±cov. association]	
Distraction capacity	N1	26	58 [7/8]	42 [3/8]	60
	N2	7	86 [3/3]	14 [0/1]	26
	N3	8	38 [1/2]	62 [3/2]	50
	N4	16	44 [5/2]	56 [2/7]	67
Reappraisal capacity	N1	26	69 [7/11]	31 [7/1]	52
	N2	7	57 [2/2]	43 [1/2]	44
	N3	8	38 [1/2]	62 [3/2]	35
	N4	16	44 [5/2]	56 [2/7]	43

*Distinctive connections for reappraisal tendency*					
Behavioral covariate	Network	Specific connections	Connection type		Specific % of total RT connections
			Inhibitory [%] and [±cov. association]	Excitatory [%] and [±cov. association]	
Reappraisal tendency	N1	25	50 [8/5]	50 [6/7]	66
	N2	26	50 [4/9]	50 [7/6]	87
	N3	21	33 [4/3]	67 [7/7]	88
	N4	21	48 [4/6]	52 [9/2]	81

*Connections modulated by distraction capacity and reappraisal capacity, and reappraisal tendency*					
Behavioral covariate	Network	Commonly modulated	Connection type		Overlap % of total RT connections
			Inhibitory [%] and [±cov. association]	Excitatory [%] and [±cov. association]	
Reappraisal tendency	N1	13	77 [6/4]	23 [3/0]	34
	N2	4	100 [2/2]	0 [0/0]	13
	N3	3	67 [1/1]	33 [1/0]	12
	N4	5	60 [1/2]	40 [1/1]	19

Abbreviations: DC, distraction capacity; I/O, input/output; RC, reappraisal capacity; RT, reappraisal tendency.

In N1 (fronto‐parietal), the primary overlapping connections associated with distraction and reappraisal capacity included connections within the frontal and parietal lobes, followed by reciprocal connections between frontal and limbic regions as well as between frontal and parietal regions. The most associations with overlap in this network were observed for connections from the left IPL to the rest of the ROIs within the network. Further overlaps were observed for associations with connections from the RACC and the LMFG to the rest of ROIs in the PFC. In N2 (fronto‐temporal), the main overlaps were observed for reciprocal connections between frontal and limbic regions and between frontal and temporal regions. Furthermore, association overlaps were observed for frontal and temporal lobes connections. Two notable reciprocal connections with overlapping associations with distraction and reappraisal capacity were between the LMFG (BA 6) and the caudate and between the LMFG (BA 6) and the LMTG. Within N3 (temporo‐limbic), primary overlap in associations with the two measures of ER capacity was observed for connections of limbic areas, that is, mainly for input and output connections of LPHG. Regarding connections within lobes, overlapping effects were observed for temporal regions. The only reciprocal connections showing an overlap for both strategies were between the LPHG and the LIOG, and the LFFA and the RFFA. Another significant connection, showing overlap in associations with distraction and reappraisal capacity, was projected from the LPHG to the MedFG. In N4 (parieto‐limbic), the main association overlap was observed for bilateral PostCG and LINS connections. Regarding connections within brain lobes, overlapping associations were also observed for connections of parietal and limbic areas. The LINS primarily received input connections from the RPostCG and projected to the bilateral PostCG in association with ER capacity.

#### 3.3.1 Strategy‐Independent Prediction of Distraction and Reappraisal Capacity

LOOCV analysis was employed to test whether the observed connections associated with distraction and reappraisal capacity significantly contributed to the prediction of the ER measures. Overlapping connectivities for distraction and reappraisal can be found in Table 3 (all connections marked in bold). Eight predictive overlapping connections were identified in N1. Most of these connections projected from frontal and parietal regions (e.g., frontal regions targeting the RMFG (BA11) and parietal regions targeting the bilateral MFG). Within N2, only one predictive connection was identified with overlap in associations with distraction and reappraisal capacity. This connection projected from the LMFG (BA6) to the LMTG. There were no overlapping predictive associations for distraction and reappraisal capacity in N3. Finally, only three overlapping connections were found to be predictive in N4, projecting from limbic to parietal regions, namely, from RPCC to RPostCG and LCun as well as from LINS to LPostCG.

### 3.4 Strategy‐Specific ER Capacity Effects

Second, we investigated connections within the four networks N1–N4 explicitly associated with distraction or reappraisal capacity. These associations are illustrated in Figure 5 for distraction capacity and in Figure 6 for reappraisal capacity (all color‐coded rectangles without dashed or dotted lines). A detailed overview of the connections within all four networks N1–N4 can be found in Table 1. The direction of the connectivity between regions (excitatory/inhibitory) is reported in Table 2.

**TABLE 2:** Associations between the distraction and reappraisal capacity and effective connectivity.

Network and connection type	Strategy	Connectivity			Covariate relation	Effect size (Hz)
		Source		Target		
Network 1	Distraction					
*Inhibition*		LMFG_BA6	→	LMFG_BA10	−	−0.31
		LMFG_BA6	→	RMFG_BA11	−	−0.72
		LMFG_BA6	→	RMFG_BA8	−	−0.42
		RMFG_BA8	→	RMFG_BA11	−	−0.60
		RMFG_BA8	→	LIPL_BA40	−	−0.22
		RMFG_BA8	→	RACC_BA23	−	−0.34
		LIPL_BA40	→	RINS_BA13	−	−0.27
		RPreCun_BA7	→	RMFG_BA11	−	−0.33
		RINS_BA13	→	RIPL_BA40	−	−0.37
		RACC_BA23	→	LMFG_BA10	−	−0.33
		LSFG_BA8	→	RIPL_BA40	+	0.38
		RMFG_BA11	→	LMFG_BA6	+	0.76
		RMFG_BA11	→	RIPL_BA40	+	0.55
		LMFG_BA6	→	RINS_BA13	+	0.76
		LIPL_BA40	→	RMFG_BA11	+	0.33
		LIPL_BA40	→	RMFG_BA8	+	0.51
		LIPL_BA40	→	RIPL_BA40	+	0.25
		RIPL_BA40	→	LMFG_BA10	+	0.37
		RIPL_BA40	→	RMFG_BA8	+	0.24
		RPreCun_BA7	→	RINS_BA13	+	0.33
		RINS_BA13	→	LIPL_BA40	+	0.36
		RINS_BA13	→	RPreCun_BA7	+	0.46
		RACC_BA23	→	LMFG_BA6	+	0.38
		RACC_BA23	→	RIPL_BA40	+	0.24
*Excitation*		RMFG_BA11	→	LMFG_BA10	−	−0.67
		LMFG_BA6	→	LSFG_BA8	−	−0.58
		LMFG_BA6	→	RIPL_BA40	−	−0.60
		RMFG_BA8	→	RIPL_BA40	−	−0.45
		RPreCun_BA7	→	LMFG_BA6	−	−0.60
		RPreCun_BA7	→	RIPL_BA40	−	−0.72
		RACC_BA23	→	RMFG_BA8	−	−0.34
		LSFG_BA8	→	LMFG_BA10	+	0.41
		LSFG_BA8	→	RMFG_BA11	+	0.52
		LSFG_BA8	→	LIPL_BA40	+	0.35
		LSFG_BA8	→	RPreCun_BA7	+	0.21
		RMFG_BA11	→	RMFG_BA8	+	0.50
		RMFG_BA11	→	LIPL_BA40	+	0.58
		RMFG_BA11	→	RPreCun_BA7	+	0.58
		RMFG_BA11	→	RACC_BA23	+	0.43
		RMFG_BA8	→	RINS_BA13	+	0.24
		LIPL_BA40	→	LSFG_BA8	+	0.48
		LIPL_BA40	→	LMFG_BA6	+	0.49
		RIPL_BA40	→	RMFG_BA11	+	0.24
Network 1	Reappraisal					
*Inhibition*		LSFG_BA8	→	RINS_BA13	−	−0.23
		LMFG_BA10	→	RMFG_BA11	−	−0.24
		RMFG_BA11	→	RPreCun_BA7	−	−0.32
		LMFG_BA6	→	LMFG_BA10	−	−0.30
		RMFG_BA8	→	LMFG_BA10	−	−0.23
		RMFG_BA8	→	RMFG_BA11	−	−0.29
		RMFG_BA8	→	RACC_BA23	−	−0.27
		LIPL_BA40	→	RPreCun_BA7	−	−0.27
		LIPL_BA40	→	RACC_BA23	−	−0.44
		RINS_BA13	→	LIPL_BA40	−	−0.33
		RINS_BA13	→	RIPL_BA40	−	−0.75
		RACC_BA23	→	LSFG_BA8	−	−0.25
		RACC_BA23	→	LMFG_BA6	−	−0.35
		RACC_BA23	→	LIPL_BA40	−	−0.25
		RMFG_BA11	→	RMFG_BA8	+	0.59
		RMFG_BA11	→	RINS_BA13	+	0.45
		LMFG_BA6	→	RINS_BA13	+	0.53
		LMFG_BA6	→	RACC_BA23	+	0.59
		RMFG_BA8	→	RINS_BA13	+	0.53
		LIPL_BA40	→	LSFG_BA8	+	0.40
		LIPL_BA40	→	RMFG_BA11	+	0.32
		LIPL_BA40	→	LMFG_BA6	+	0.53
		LIPL_BA40	→	RMFG_BA8	+	0.69
		LIPL_BA40	→	RIPL_BA40	+	0.27
		LIPL_BA40	→	RINS_BA13	+	0.35
		RIPL_BA40	→	LSFG_BA8	+	0.26
		RIPL_BA40	→	LMFG_BA10	+	0.58
		RIPL_BA40	→	LMFG_BA6	+	0.37
		RIPL_BA40	→	RPreCun_BA7	+	0.41
		RPreCun_BA7	→	RINS_BA13	+	0.61
*Excitation*		LSFG_BA8	→	LMFG_BA6	−	−0.60
		LSFG_BA8	→	RMFG_BA8	−	−0.64
		LSFG_BA8	→	LIPL_BA40	−	−0.28
		LMFG_BA10	→	RIPL_BA40	−	−0.42
		LMFG_BA10	→	RACC_BA23	−	−0.35
		RMFG_BA11	→	RACC_BA23	−	−0.29
		LMFG_BA6	→	LSFG_BA8	−	−0.26
		LMFG_BA6	→	RMFG_BA11	−	−0.51
		RIPL_BA40	→	RINS_BA13	−	−0.51
		RPreCun_BA7	→	RIPL_BA40	−	−0.45
		RACC_BA23	→	LMFG_BA10	−	−0.26
		RACC_BA23	→	RMFG_BA8	−	−0.36
		LSFG_BA8	→	RMFG_BA11	+	0.47
		LSFG_BA8	→	RACC_BA23	+	0.23
		LMFG_BA10	→	LMFG_BA6	+	0.37
		LMFG_BA6	→	RPreCun_BA7	+	0.34
		RIPL_BA40	→	LIPL_BA40	+	0.36
		RIPL_BA40	→	RACC_BA23	+	0.35
		RINS_BA13	→	LSFG_BA8	+	0.35
		RPreCun_BA7	→	LIPL_BA40	+	0.61
Network 2	Distraction					
*Inhibition*		LSFG_BA6	→	LIFG_BA47	−	−0.52
		LSFG_BA6	→	LMTG	−	−0.31
		LSFG_BA6	→	LCaudate	−	−0.20
		LSFG_BA9	→	LMTG	−	−0.23
		LMFG_BA6	→	LCaudate	−	−0.26
		LIFG_BA47	→	LSFG_BA6	−	−0.25
		RIFG_BA47	→	LSTG_BA39	−	−0.25
		LSTG_BA39	→	LMTG	−	−0.29
		LMTG	→	LMFG_BA6	−	−0.33
		LCaudate	→	LIFG_BA47	−	−0.63
		LCaudate	→	RIFG_BA47	−	−0.41
		LCaudate	→	LSFG_BA9	+	0.48
		LCaudate	→	LMFG_BA6	+	0.49
		LSFG_BA6	→	LSTG_BA39	+	0.31
		LMFG_BA6	→	LMTG	+	0.44
		LSTG_BA39	→	RIFG_BA47	+	0.27
*Excitation*		LSFG_BA6	→	RIFG_BA47	−	−0.70
		LIFG_BA47	→	LMFG_BA6	−	−0.27
		LIFG_BA47	→	RIFG_BA47	−	−0.26
		LMTG	→	LIFG_BA47	−	−0.54
		LSFG_BA6	→	LSFG_BA9	+	0.88
		LSFG_BA9	→	LIFG_BA47	+	0.45
		LSFG_BA9	→	RIFG_BA47	+	0.36
		LSFG_BA9	→	LCaudate	+	0.25
		LMFG_BA6	→	RIFG_BA47	+	0.30
		RIFG_BA47	→	LSFG_BA6	+	0.36
		RIFG_BA47	→	LIFG_BA47	+	0.28
Network 2	Reappraisal					
*Inhibition*		LSFG_BA9	→	LSFG_BA6	−	−0.26
		LSTG_BA39	→	LMTG	−	−0.31
		LMTG	→	LMFG_BA6	−	−0.61
		LCaudate	→	LSFG_BA6	−	−0.51
		LCaudate	→	LMTG	−	−0.34
		LSTG_BA39	→	LSFG_BA6	+	0.35
		LSTG_BA39	→	LIFG_BA47	+	0.56
		LSTG_BA39	→	RIFG_BA47	+	0.30
		LMTG	→	LSTG_BA39	+	0.32
		LCaudate	→	LMFG_BA6	+	0.34
		LCaudate	→	LSTG_BA39	+	0.36
*Excitation*		LMFG_BA6	→	LCaudate	−	−0.43
		RIFG_BA47	→	LSFG_BA9	−	−0.40
		LSFG_BA6	→	LSFG_BA9	+	0.53
		LMFG_BA6	→	LMTG	+	0.85
		RIFG_BA47	→	LMTG	+	0.36
Network 3	Distraction					
*Inhibition*		LAMY	→	RThal	−	−0.61
		RAMY	→	LPHG_BA27	−	−0.17
		LPHG_BA27	→	LIOG_BA19	+	0.60
		LPHG_BA27	→	RThal	+	0.41
		LPHG_BA27	→	LAMY	+	0.36
*Excitation*		LFFA_BA37	→	RFFA_BA37	−	−0.70
		LFFA_BA37	→	LIOG_BA19	−	−0.47
		LIOG_BA19	→	LPHG_BA27	−	−0.24
		RThal	→	LIOG_BA19	−	−0.49
		LFFA_BA37	→	RThal	+	0.35
		LFFA_BA37	→	LPHG_BA27	+	0.38
		RFFA_BA37	→	LFFA_BA37	+	0.50
		LPHG_BA27	→	MedFG_BA10	+	0.46
		LAMY	→	LIOG_BA19	+	0.26
		LAMY	→	LPHG_BA27	+	0.29
		RAMY	→	RFFA_BA37	+	0.22
Network 3	Reappraisal					
*Inhibition*		LIOG_BA19	→	RAMY	−	−0.25
		LPHG_BA27	→	RAMY	−	−0.39
		LAMY	→	RThal	−	−0.52
		MedFG_BA10	→	RThal	+	0.20
		MedFG_BA10	→	RAMY	+	0.23
		LFFA_BA37	→	LAMY	+	0.35
		RFFA_BA37	→	MedFG_BA10	+	0.40
		RFFA_BA37	→	RThal	+	0.41
		LIOG_BA19	→	LFFA_BA37	+	0.49
		LIOG_BA19	→	RFFA_BA37	+	0.46
		LPHG_BA27	→	LIOG_BA19	+	0.81
		LPHG_BA27	→	RThal	+	0.69
		RAMY	→	RThal	+	0.35
*Excitation*		LFFA_BA37	→	RFFA_BA37	−	−0.37
		LFFA_BA37	→	LIOG_BA19	−	−0.37
		LIOG_BA19	→	LPHG_BA27	−	−0.36
		RThal	→	LPHG_BA27	−	−0.35
		RAMY	→	MedFG_BA10	−	−0.28
		RFFA_BA37	→	LFFA_BA37	+	0.43
		LPHG_BA27	→	MedFG_BA10	+	0.70
		LPHG_BA27	→	RFFA_BA37	+	0.30
		RThal	→	LAMY	+	0.47
		LAMY	→	LFFA_BA37	+	0.21
Network 4	Distraction					
*Inhibition*		LPostCG_BA2	→	LMOG_BA19	−	−0.35
		RPostCG_BA2	→	LCun_BA18	−	−0.27
		RPostCG_BA2	→	LINS_BA13	−	−0.27
		LSPL_BA7	→	LPostCG_BA2	−	−0.46
		LCun_BA18	→	RPCC_BA30	−	−0.35
		LINS_BA13	→	LCun_BA18	−	−0.33
		RPostCG_BA2	→	RPCC_BA30	+	0.21
		RPreCun_BA19	→	LPostCG_BA2	+	0.36
		RThal	→	LPostCG_BA2	+	0.48
*Excitation*		LPostCG_BA2	→	LSPL_BA7	−	−0.28
		LPostCG_BA2	→	RThal	−	−0.29
		LSPL_BA7	→	RPreCun_BA19	−	−0.30
		RPCC_BA30	→	LPostCG_BA2	−	−0.27
		RPCC_BA30	→	RPostCG_BA2	−	−0.51
		RPostCG_BA2	→	LSPL_BA7	+	0.20
		RPostCG_BA2	→	RThal	+	0.32
		RPreCun_BA19	→	RPostCG_BA2	+	0.47
		RPreCun_BA19	→	LSPL_BA7	+	0.39
		RPreCun_BA19	→	RThal	+	0.32
		LMOG_BA19	→	LPostCG_BA2	+	0.26
		LINS_BA13	→	LPostCG_BA2	+	0.44
		LINS_BA13	→	RPostCG_BA2	+	0.71
		LINS_BA13	→	RPCC_BA30	+	0.35
		RPCC_BA30	→	LCun_BA18	+	0.41
Network 4	Reappraisal					
*Inhibition*		LPostCG_BA2	→	LMOG_BA19	−	−0.25
		RPostCG_BA2	→	RPreCun_BA19	−	−0.18
		RPostCG_BA2	→	LINS_BA13	−	−0.28
		LSPL_BA7	→	LPostCG_BA2	−	−0.57
		LSPL_BA7	→	RPostCG_BA2	−	−0.22
		RPreCun_BA19	→	RPCC_BA30	−	−0.63
		LMOG_BA19	→	RThal	−	−0.35
		LCun_BA18	→	RPCC_BA30	−	−0.46
		LINS_BA13	→	LCun_BA18	−	−0.50
		RPCC_BA30	→	LINS_BA13	−	−0.25
		RThal	→	RPCC_BA30	−	−0.57
		RThal	→	LPostCG_BA2	+	0.32
		RPostCG_BA2	→	RPCC_BA30	+	0.23
		LSPL_BA7	→	LMOG_BA19	+	0.49
		LSPL_BA7	→	RPCC_BA30	+	0.28
		LCun_BA18	→	RPostCG_BA2	+	0.38
		LCun_BA18	→	LINS_BA13	+	0.45
		RPCC_BA30	→	LMOG_BA19	+	0.31
		RPCC_BA30	→	RThal	+	0.30
		RThal	→	LMOG_BA19	+	0.36
		RThal	→	LCun_BA18	+	0.29
		RThal	→	LINS_BA13	+	0.33
*Excitation*		LPostCG_BA2	→	LSPL_BA7	−	−0.29
		LMOG_BA19	→	LSPL_BA7	−	−0.24
		LMOG_BA19	→	RPCC_BA30	−	−0.44
		LINS_BA13	→	RThal	−	−0.23
		RPCC_BA30	→	RPostCG_BA2	−	−0.58
		RPCC_BA30	→	LSPL_BA7	−	−0.33
		LPostCG_BA2	→	RPostCG_BA2	+	0.48
		LPostCG_BA2	→	LINS_BA13	+	0.37
		RPostCG_BA2	→	LSPL_BA7	+	0.21
		RPostCG_BA2	→	RThal	+	0.20
		LMOG_BA19	→	LPostCG_BA2	+	0.21
		LINS_BA13	→	LPostCG_BA2	+	0.44
		LINS_BA13	→	RPostCG_BA2	+	0.59
		LINS_BA13	→	RPCC_BA30	+	0.33
		RPCC_BA30	→	LCun_BA18	+	0.32

Abbreviations: BA, Brodmann area; LAMY, left amygdala; LCaudate, left caudate; LCun, left cuneus; LFFA, left fusiform face area; LIFG, left inferior frontal gyrus; LINS, left insula; LIPL, left inferior parietal lobule; LMFG, left middle frontal gyrus; LMOG, left middle occipital gyrus; LMTG, left middle temporal gyrus; LPostCG, left postcentral gyrus; LPHG, left parahippocampal gyrus; LSFG, left superior frontal gyrus; LSPL, left superior parietal lobule; LSTG, left superior temporal gyrus; MedFG, medial frontal gyrus; RACC, right anterior cingulate cortex; RAMY, right amygdala; RFFA, right fusiform face area; RIFG, right inferior frontal gyrus; RINS, right insula; RIPL, right inferior parietal lobule; RMFG, right middle frontal gyrus; RPCC, right posterior cingulate; RPostCG, right postcentral gyrus; RPreCun, right precuneus; RThal, right thalamus.

Consistent with the view that frontoparietal control architecture supports regulatory ability, individual differences in N1 coupling at rest were associated with implementation capacity. We stress that this association is correlational and pertains to baseline network organization, not to state‐evoked mechanisms. Within network N1, both distraction capacity (Figure 5A and Table 1) and reappraisal capacity (Figure 6A) showed associations with reciprocal connections between frontal and parietal regions. For both ER strategies, ER capacity modulated connections from frontal to parietal and from parietal to frontal regions. Furthermore, distraction capacity was positively associated with two connections from limbic to parietal regions. Most associations were observed for distraction capacity with connections from and to the RIPL. In contrast, in N1, specific effects for reappraisal capacity were observed for negative associations for limbic regions that projected to frontal and parietal regions. In addition, mostly positive associations of reappraisal capacity with connections from frontal regions to limbic regions and negative associations with connections from parietal regions to limbic regions were observed. Overall, reappraisal capacity showed the most associations with input/output connections of LSFG and RIPL. Associations with connections within brain lobes (e.g., frontal‐to‐frontal) were mainly observed for the frontal lobe, for example, distraction capacity with connectivity from RMFG (BA11) to LMFG (BA6) and reappraisal capacity with connectivity from LMFG (BA10) to LMFG (BA6). In network N2 (fronto‐temporal), distraction capacity was linked with more specific input/output connections than reappraisal capacity (Figures 5B and 6B and Table 1). Reciprocal connections between frontal and temporal regions were associated with distraction and reappraisal capacity, primarily negative associations for distraction capacity and positive associations for reappraisal capacity. Frontal regions had the most input/output connections related to distraction and reappraisal capacity. The regions with the most input/output connections related to distraction capacity were LSFG and bilateral IFG. Specifically, for the ROIs within the LSFG (BA6, BA9), projections to all other lobes of the network were associated with distraction capacity. Furthermore, distraction capacity was explicitly associated with connections from the caudate to nearly all PFC regions. In contrast, for reappraisal capacity, most associations to input/output connections were observed for LSTG and LMTG. Regarding connectivity within brain lobes, frontal regions had the most input/output connections showing a negative association with reappraisal and distraction capacity. Within N3 (temporo‐limbic), distraction capacity was associated with fewer specific input/output connections than reappraisal capacity (Figures 5C and 6C and Table 1). Regarding connectivity within brain lobes, the limbic regions had opposite associations with the two capacity measures—being positively associated with distraction capacity and negatively with reappraisal capacity. The brain region with the most input/output connections being associated with distraction capacity was LPHG, with input connections from the bilateral amygdalae. Reappraisal capacity, on the other hand, showed most associations with input/output connections of the RThal and LAMY. Notably, in association with reappraisal capacity, there was a reciprocal connection between the MedFG and RAMY, with connections from the MedFG to the RAMY being positively associated and connections from RAMY to MedFG being negatively associated with reappraisal capacity. In addition, associations specific to reappraisal capacity were observed for connections projecting from RAMY and MedFG to the RThal. In N4 (parieto‐limbic), within the parietal lobe, connections were associated with distraction and reappraisal capacity (Figures 5D and 6D and Table 1). Of note, an opposite effect was observed for reciprocal connections between the parietal and the occipital regions: Connections from the parietal to the occipital areas were negatively associated with distraction, as well as negatively and positively with reappraisal capacity. In contrast, the connections projecting from the occipital to the parietal region were positively related to distraction capacity and negatively to reappraisal capacity. Most associations with input/output connections were observed for distraction capacity for the LPostCG. Most associations with input/output connections were observed for reappraisal capacity for RPCC.

#### 3.4.1 Strategy‐Dependent Prediction for Distraction and Reappraisal Capacity

**FIGURE 7:**
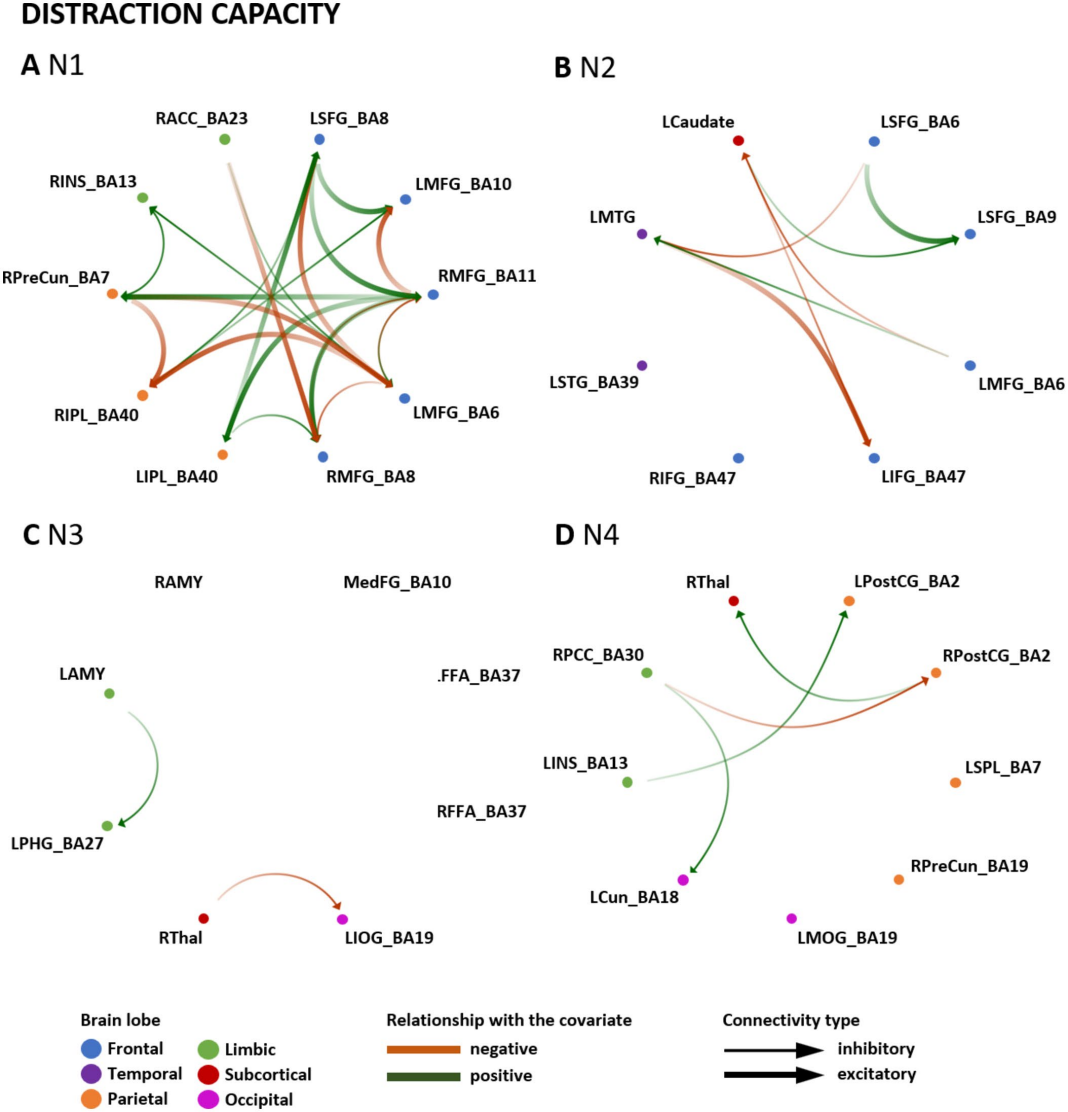
Prediction of distraction capacity by effective connectivity in networks N1–N4. LOOCV for the prediction of distraction capacity by effective connectivity in networks N1–N4 with significant effect sizes (*p* < 0.05). The green and red arrows indicate the association of distraction capacity with EC in each network (green: positive association with ER measure; red: negative association). The color saturation shows the direction of the connectivity (i.e., source to target: from less to more saturated with an arrow). The thickness of the line (i.e., thin or thick) indicates whether the connection is inhibitory or excitatory. Brain regions are organized from cortical to limbic areas. BA, Brodmann area; LAMY, left amygdala; LCaudate, left caudate; LCun, left cuneus; LFFA, left fusiform face area; LIFG, left inferior frontal gyrus; LINS, left insula; LIOG, left inferior occipital gyrus; LIPL, left inferior parietal lobule; LMFG, left middle frontal gyrus; LMOG, left middle occipital gyrus; LMTG, left middle temporal gyrus; LPHG, left parahippocampal gyrus; LPostCG, left postcentral gyrus; LSFG, left superior frontal gyrus; LSPL, left superior parietal lobule; LSTG, left superior temporal gyrus; MedFG, medial frontal gyrus; RACC, right anterior cingulate cortex; RAMY, right amygdala; RFFA, right fusiform face area; RIFG, right inferior frontal gyrus; RINS, right insula; RIPL, right inferior parietal lobule; RMFG, right middle frontal gyrus; RPCC, right posterior cingulate; RPostCG, right postcentral gyrus; RPreCun, right precuneus; RThal, right thalamus.

**FIGURE 8:**
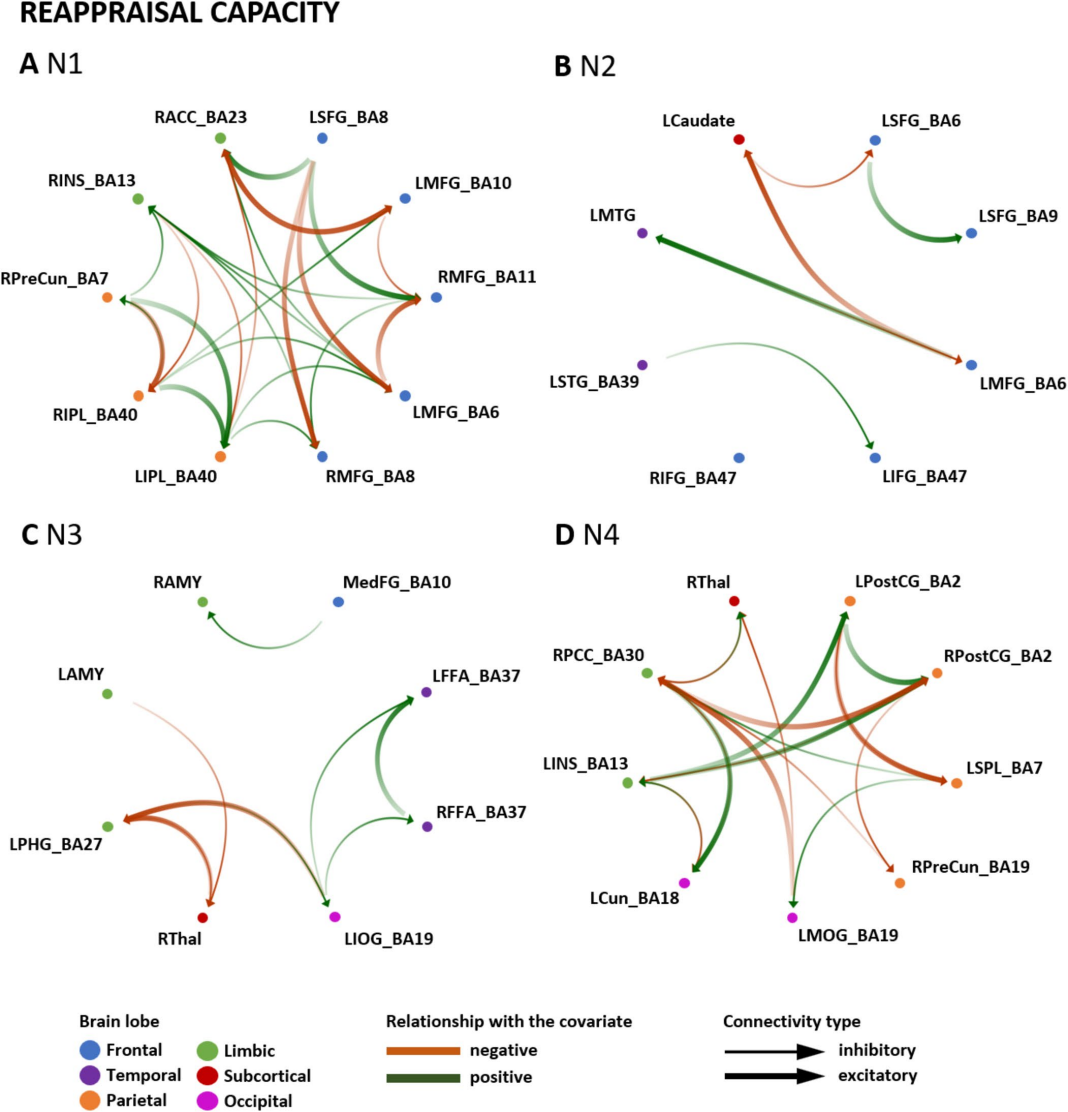
Prediction of reappraisal capacity by effective connectivity in networks N1–N4. LOOCV for the prediction of reappraisal capacity by effective connectivity in networks N1–N4 with significant effect sizes (*p* < 0.05). The green and red arrows indicate the association of distraction capacity with EC in each network (green: positive association with ER measure; red: negative association). The color saturation shows the direction of the connectivity (i.e., source to target: from less to more saturated with an arrow). The thickness of the line (i.e., thin or thick) indicates whether the connection is inhibitory or excitatory. Brain regions are organized from cortical to limbic areas. BA, Brodmann area; LAMY, left amygdala; LCaudate, left caudate; LCun, left cuneus; LFFA, left fusiform face area; LIFG, left inferior frontal gyrus; LINS, left insula; LIOG, left inferior occipital gyrus; LIPL, left inferior parietal lobule; LMFG, left middle frontal gyrus; LMOG, left middle occipital gyrus; LMTG, left middle temporal gyrus; LPHG, left parahippocampal gyrus; LPostCG, left postcentral gyrus; LSFG, left superior frontal gyrus; LSPL, left superior parietal lobule; LSTG, left superior temporal gyrus; MedFG, medial frontal gyrus; RACC, right anterior cingulate cortex; RAMY, right amygdala; RFFA, right fusiform face area; RIFG, right inferior frontal gyrus; RINS, right insula; RIPL, right inferior parietal lobule; RMFG, right middle frontal gyrus; RPCC, right posterior cingulate; RPostCG, right postcentral gyrus; RPreCun, right precuneus; RThal, right thalamus.

LOOCV was conducted to identify which connections were specifically contributing to the prediction of either distraction or reappraisal capacity (i.e., bold connections in Table 3). The results of the LOOCV are illustrated in Figures 7 and 8 and summarized in Table 3.

**TABLE 3:** Results of the leave‐one‐out cross‐validation analyses for distraction and reappraisal capacity.

Network and connection type	Strategy	Connectivity			*r*‐value	*p*‐value
		Source		Target		
Network 1	Distraction					
*Inhibition*		LSFG_BA8	→	RIPL_BA40	0.33	0.02
		RMFG_BA11	→	LMFG_BA6	0.37	0.01
		LMFG_BA6	→	RMFG_BA11	0.43	**0.00**
		LMFG_BA6	→	RMFG_BA8	0.28	0.04
		LMFG_BA6	→	RINS_BA13	0.48	**0.00**
		RMFG_BA8	→	RMFG_BA11	0.42	0.00
		LIPL_BA40	→	RMFG_BA8	0.32	**0.02**
		RIPL_BA40	→	LMFG_BA10	0.29	**0.03**
		RPreCun_BA7	→	RINS_BA13	0.27	**0.04**
		RINS_BA13	→	RPreCun_BA7	0.32	0.02
		RACC_BA23	→	LMFG_BA6	0.30	0.03
*Excitation*		LSFG_BA8	→	LMFG_BA10	0.37	0.01
		LSFG_BA8	→	RMFG_BA11	0.33	**0.02**
		RMFG_BA11	→	LMFG_BA10	0.31	0.03
		RMFG_BA11	→	RMFG_BA8	0.34	**0.02**
		RMFG_BA11	→	LIPL_BA40	0.35	0.01
		RMFG_BA11	→	RPreCun_BA7	0.29	0.04
		LMFG_BA6	→	LSFG_BA8	0.34	0.02
		LMFG_BA6	→	RIPL_BA40	0.34	0.02
		LIPL_BA40	→	LSFG_BA8	0.28	0.04
		RPreCun_BA7	→	LMFG_BA6	0.36	0.01
		RPreCun_BA7	→	RIPL_BA40	0.36	**0.01**
		RACC_BA23	→	RMFG_BA8	0.28	0.04
Network 1	Reappraisal					
*Inhibition*		LMFG_BA10	→	RMFG_BA11	0.27	0.05
		RMFG_BA11	→	RMFG_BA8	0.42	**0.00**
		RMFG_BA11	→	RINS_BA13	0.29	0.03
		LMFG_BA6	→	RINS_BA13	0.29	**0.04**
		LMFG_BA6	→	RACC_BA23	0.38	0.01
		RMFG_BA8	→	RINS_BA13	0.36	0.01
		LIPL_BA40	→	LMFG_BA6	0.31	0.03
		LIPL_BA40	→	RMFG_BA8	0.34	**0.01**
		LIPL_BA40	→	RACC_BA23	0.33	0.02
		RIPL_BA40	→	LMFG_BA6	0.30	0.03
		RIPL_BA40	→	RPreCun_BA7	0.30	0.03
		RIPL_BA40	→	LMFG_BA10	0.35	**0.01**
		RPreCun_BA7	→	RINS_BA13	0.33	**0.02**
		RINS_BA13	→	LIPL_BA40	0.29	0.04
		RINS_BA13	→	RIPL_BA40	0.38	0.01
*Excitation*		LSFG_BA8	→	RMFG_BA11	0.33	0.02
		LSFG_BA8	→	LMFG_BA6	0.33	0.02
		LSFG_BA8	→	RMFG_BA8	0.30	**0.03**
		LSFG_BA8	→	RACC_BA23	0.30	0.03
		LMFG_BA10	→	RACC_BA23	0.33	0.02
		LMFG_BA6	→	RMFG_BA11	0.35	**0.01**
		RIPL_BA40	→	LIPL_BA40	0.30	0.03
		RPreCun_BA7	→	LIPL_BA40	0.40	0.01
		RPreCun_BA7	→	RIPL_BA40	0.28	**0.04**
		RACC_BA23	→	LMFG_BA10	0.29	0.04
Network 2	Distraction					
*Inhibition*		LSFG_BA6	→	LMTG	0.42	0.00
		LMFG_BA6	→	LMTG	0.28	**0.04**
		LMFG_BA6	→	LCaudate	0.27	**0.05**
		LCaudate	→	LSFG_BA9	0.31	0.03
		LCaudate	→	LIFG_BA47	0.32	0.02
*Excitation*		LSFG_BA6	→	LSFG_BA9	0.33	**0.02**
		LMTG	→	LIFG_BA47	0.29	0.04
Network 2	Reappraisal					
*Inhibition*		LSTG_BA39	→	LIFG_BA47	0.32	0.02
		LMTG	→	LMFG_BA6	0.30	0.03
		LCaudate	→	LSFG_BA6	0.34	0.02
*Excitation*		LSFG_BA6	→	LSFG_BA9	0.35	**0.01**
		LMFG_BA6	→	LMTG	0.41	**0.00**
		LMFG_BA6	→	LCaudate	0.33	**0.02**
Network 3	Distraction					
*Excitation*		RThal	→	LIOG_BA19	0.36	0.01
		LAMY	→	LPHG_BA27	0.40	0.01
Network 3	Reappraisal					
*Inhibition*		MedFG_BA10	→	RAMY	0.29	0.03
		LIOG_BA19	→	LFFA_BA37	0.28	0.04
		LIOG_BA19	→	RFFA_BA37	0.34	0.02
		LPHG_BA27	→	LIOG_BA19	0.42	0.00
		LPHG_BA27	→	RThal	0.37	0.01
		LAMY	→	RThal	0.27	0.05
*Excitation*		RFFA_BA37	→	LFFA_BA37	0.29	0.03
		LIOG_BA19	→	LPHG_BA27	0.35	0.01
		RThal	→	LPHG_BA27	0.29	0.04
Network 4	Distraction					
*Excitation*		RPostCG_BA2	→	RThal	0.33	0.02
		LINS_BA13	→	LPostCG_BA2	0.27	**0.05**
		RPCC_BA30	→	RPostCG_BA2	0.29	**0.03**
		RPCC_BA30	→	LCun_BA18	0.39	**0.01**
Network 4	Reappraisal					
*Inhibition*		RPostCG_BA2	→	RPreCun_BA19	0.27	0.05
		RPostCG_BA2	→	LINS_BA13	0.27	0.04
		RPreCun_BA19	→	RPCC_BA30	0.39	0.01
		LSPL_BA7	→	LPostCG_BA2	0.29	0.04
		LSPL_BA7	→	LMOG_BA19	0.33	0.02
		LSPL_BA7	→	RPCC_BA30	0.28	0.04
		LMOG_BA19	→	RThal	0.37	0.01
		LCun_BA18	→	LINS_BA13	0.27	0.05
		LCun_BA18	→	RPCC_BA30	0.28	0.04
		LINS_BA13	→	LCun_BA18	0.34	0.02
		RPCC_BA30	→	RThal	0.31	0.03
		RThal	→	RPCC_BA30	0.31	0.02
*Excitation*		LPostCG_BA2	→	LSPL_BA7	0.30	0.03
		LPostCG_BA2	→	RPostCG_BA2	0.29	0.03
		LMOG_BA19	→	RPCC_BA30	0.32	0.02
		LINS_BA13	→	LPostCG_BA2	0.32	**0.02**
		LINS_BA13	→	RPostCG_BA2	0.28	0.04
		RPCC_BA30	→	RPostCG_BA2	0.46	**0.00**
		RPCC_BA30	→	LCun_BA18	0.30	**0.03**

*Note:* Bold values indicate overlapping connectivities between the covariates.

Abbreviations: Connections with the predictive ability for both distraction and reappraisal *capacity* are marked in bold. BA, Brodmann area; LAMY, left amygdala; LCaudate, left caudate; LCun, left cuneus; LFFA, left fusiform face area; LIFG, left inferior frontal gyrus; LINS, left insula; LIPL, left inferior parietal lobule; LMFG, left middle frontal gyrus; LMOG, left middle occipital gyrus; LMTG, left middle temporal gyrus; LPostCG, left postcentral gyrus; LPHG, left parahippocampal gyrus; LSFG, left superior frontal gyrus; LSPL, left superior parietal lobule; LSTG, left superior temporal gyrus; MedFG, medial frontal gyrus; RACC, right anterior cingulate cortex; RAMY, right amygdala; RFFA, right fusiform face area; RIFG, right inferior frontal gyrus; RINS, right insula; RIPL, right inferior parietal lobule; RMFG, right middle frontal gyrus; RPCC, right posterior cingulate; RPostCG, right postcentral gyrus; RPreCun, right precuneus; RThal, right thalamus.

In N1 (fronto‐parietal), more predictive connections were observed for reappraisal capacity than for distraction capacity. Distraction capacity‐specific predictive connections were identified projecting from the frontal regions to the bilateral MFG and IPL. In contrast, connections predictive of reappraisal capacity projected from the frontal and parietal regions and targeted the bilateral MFG and limbic regions. Across both reappraisal and distraction capacity, the LMFG (BA6) contributed the most significant number of predictive edges. For reappraisal, LMFG (BA6) showed directed influence on the RMFG (BA11), the RINS, and RACC. For distraction, LMFG (BA6) influenced two right MFG clusters (BA11, BA8), the LSFG, the RINS, and the RIPL. For N2 (fronto‐temporal), four specific connections were observed in relation to distraction and three associated with reappraisal capacity. The LSFG (BA6) and LMFG (BA6), projecting to the LMTG, LSFG (BA9) and LCaudate, the LCaudate targeting LSFG (BA9) and LIFG (BA47), and the LMTG targeting LIFG (BA47) were the connections predictive of distraction capacity. Regarding reappraisal capacity, frontal regions (LSFG and LMFG (BA6)) had predictive connections projecting to LSFG (BA9), LMTG, and LCaudate. Further, the LMTG and LCaudate showed predictive connections targeting LMFG (BA6) and LSFG (BA6), respectively. LMFG (BA6) represented the region with the highest number of predictive connections. When looking at N3 (temporo‐limbic), only two connections could be identified as predictive of distraction capacity (i.e., RThal projecting to LIOG, and LAMY to LPHG). In contrast, nine connections were observed to be predictive of reappraisal capacity. The regions with the most predictive connections associated with reappraisal capacity were the LIOG, followed by the LPHG, with a reciprocal connection between them. Another reciprocal connection was identified between RThal and LPHG, which was predictive of reappraisal capacity. Within N4 (parieto‐limbic), more predictive connections were identified for reappraisal than distraction capacity. For distraction capacity, only one specific connection was observed, namely from RPostCG to RThal. For reappraisal capacity, most predictive connections were within parietal regions (e.g., a reciprocal predictive connection between the LSPL and LPostCG). In addition, the limbic areas had predictive input connections from regions of all other lobes in the network (e.g., RPCC receiving input from LSPL, RPreCun, LCun, LMOG, and RThal).

### 3.5 Reappraisal Tendency Effects

**FIGURE 9:**
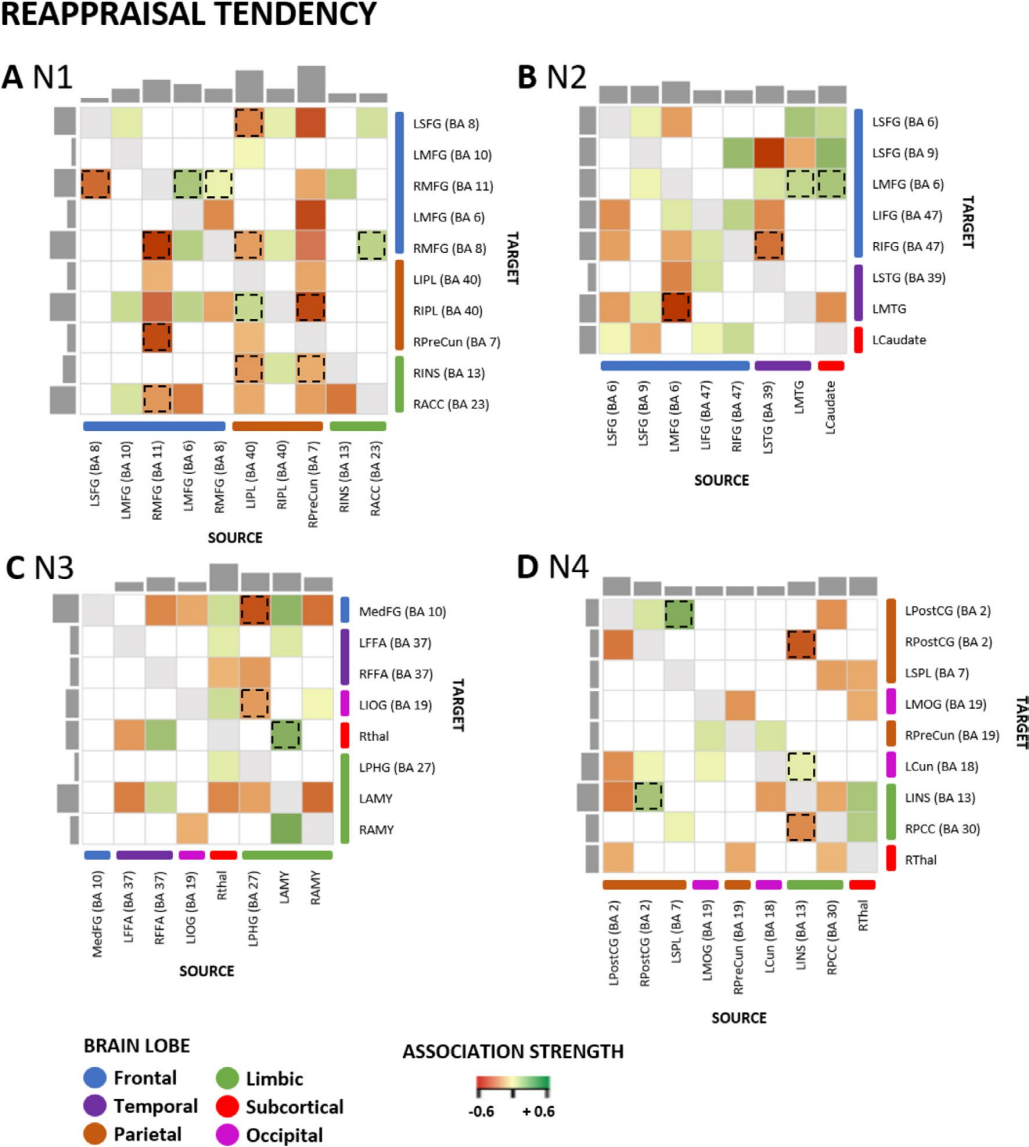
Reappraisal tendency and effective connectivity within ER networks N1–N4. Association between reappraisal tendency for high‐intensity negative stimuli and EC within each network (N1–N4). Association strength: The colors in the heat map characterize the association of the ER variable with the EC, with green indicating a positive and red a negative association measured in Hertz (Hz). Dashed lines demonstrate the overlapping connections between distraction and reappraisal capacity, and reappraisal tendency. Only associations exceeding a conservative threshold of posterior probability > 0.99 are reported. Matrix columns: source regions; rows: target regions. The number of outputs and inputs of specific regions is illustrated with the grey bars on the side (inputs) and top (outputs) of the matrix. BA, Brodmann area; LAMY, left amygdala; LCaudate, left caudate; LCun, left cuneus; LFFA, left fusiform face area; LIFG, left inferior frontal gyrus; LINS, left insula; LIOG, left inferior occipital gyrus; LIPL, left inferior parietal lobule; LMFG, left middle frontal gyrus; LMOG, left middle occipital gyrus; LMTG, left middle temporal gyrus; LPHG, left parahippocampal gyrus; LPostCG, left postcentral gyrus; LSFG, left superior frontal gyrus; LSPL, left superior parietal lobule; LSTG, left superior temporal gyrus; MedFG, medial frontal gyrus; RACC, right anterior cingulate cortex; RAMY, right amygdala; RFFA, right fusiform face area; RIFG, right inferior frontal gyrus; RINS, right insula; RIPL, right inferior parietal lobule; RMFG, right middle frontal gyrus; RPCC, right posterior cingulate; RPostCG, right postcentral gyrus; RPreCun, right precuneus; RThal, right thalamus.

Third, we were interested in the associations between effective connectivity and ER tendency. The findings regarding associations between reappraisal tendency and effective connectivity among the ROIs of the four ER networks are illustrated in Figure 9 and summarized in Table 4. All connections specifically associated with reappraisal tendency (illustrated as color‐coded rectangles without dashed or dotted lines in Figure 9), as well as the connections modulated by distraction capacity, reappraisal capacity, and reappraisal tendency (color‐coded rectangles with dashed or dotted lines in Figure 9), are reported in Table 1.

**TABLE 4:** Associations between the reappraisal tendency and effective connectivity.

Network and connection type	Connectivity			Covariate relation	Effect size (Hz)
	Source		Target		
Network 1					
*Inhibition*	RMFG_BA11	→	RMFG_BA8	−	−0.45
	RMFG_BA11	→	LIPL_BA40	−	−0.12
	RMFG_BA11	→	RPreCun_BA7	−	−0.40
	LMFG_BA6	→	RACC_BA23	−	−0.27
	LIPL_BA40	→	LSFG_BA8	−	−0.26
	LIPL_BA40	→	RMFG_BA8	−	−0.18
	LIPL_BA40	→	RPreCun_BA7	−	−0.11
	LIPL_BA40	→	RINS_BA13	−	−0.19
	LIPL_BA40	→	RACC_BA23	−	−0.15
	RPreCun_BA7	→	RMFG_BA8	−	−0.62
	RPreCun_BA7	→	RINS_BA13	−	−0.14
	RPreCun_BA7	→	RACC_BA23	−	−0.18
	RINS_BA13	→	RACC_BA23	−	−0.28
	RMFG_BA11	→	RIPL_BA40	−	−0.58
	LMFG_BA6	→	RMFG_BA11	+	0.27
	LMFG_BA6	→	RMFG_BA8	+	0.24
	RMFG_BA8	→	RMFG_BA11	+	0.13
	LIPL_BA40	→	LMFG_BA10	+	0.11
	LIPL_BA40	→	RIPL_BA40	+	0.21
	RIPL_BA40	→	LSFG_BA8	+	0.13
	RIPL_BA40	→	RMFG_BA8	+	0.15
	RACC_BA23	→	RMFG_BA8	+	0.22
*Excitation*	LSFG_BA8	→	RMFG_BA11	−	−0.32
	RMFG_BA11	→	RACC_BA23	−	−0.20
	RMFG_BA8	→	LMFG_BA6	−	−0.23
	RMFG_BA8	→	RIPL_BA40	−	−0.16
	RPreCun_BA7	→	LSFG_BA8	−	−0.51
	RPreCun_BA7	→	RMFG_BA11	−	−0.15
	RPreCun_BA7	→	LMFG_BA6	−	−0.49
	RPreCun_BA7	→	LIPL_BA40	−	−0.16
	RPreCun_BA7	→	RIPL_BA40	−	−0.40
	LMFG_BA10	→	LSFG_BA8	+	0.14
	LMFG_BA10	→	RIPL_BA40	+	0.20
	LMFG_BA10	→	RACC_BA23	+	0.17
	LMFG_BA6	→	RIPL_BA40	+	0.22
	RIPL_BA40	→	RINS_BA13	+	0.16
	RINS_BA13	→	RMFG_BA11	+	0.24
	RACC_BA23	→	LSFG_BA8	+	0.18
Network 2					
*Inhibition*	LSFG_BA6	→	LMTG	−	−0.18
	LMFG_BA6	→	LMTG	−	−0.44
	LSTG_BA39	→	LSFG_BA9	−	−0.43
	LSTG_BA39	→	LIFG_BA47	−	−0.23
	LSTG_BA39	→	RIFG_BA47	−	−0.30
	LCaudate	→	LMTG	−	−0.22
	LSFG_BA9	→	LSFG_BA6	+	0.12
	LSFG_BA9	→	LMFG_BA6	+	0.11
	LSFG_BA9	→	LMTG	+	0.14
	LMFG_BA6	→	LIFG_BA47	+	0.15
	LIFG_BA47	→	LCaudate	+	0.11
	LSTG_BA39	→	LMFG_BA6	+	0.16
	LMTG	→	LSFG_BA6	+	0.28
	LMTG	→	LMFG_BA6	+	0.21
	LCaudate	→	LSFG_BA6	+	0.21
	LCaudate	→	LSFG_BA9	+	0.33
	LCaudate	→	LMFG_BA6	+	0.27
*Excitation*	LSFG_BA6	→	LIFG_BA47	−	−0.22
	LSFG_BA6	→	RIFG_BA47	−	−0.17
	LSFG_BA9	→	LCaudate	−	−0.15
	LMFG_BA6	→	LSFG_BA6	−	−0.18
	LMFG_BA6	→	RIFG_BA47	−	−0.16
	LMFG_BA6	→	LSTG_BA39	−	−0.25
	LMTG	→	LSFG_BA9	−	−0.14
	LSFG_BA6	→	LCaudate	+	0.11
	LIFG_BA47	→	RIFG_BA47	+	0.17
	LIFG_BA47	→	LSTG_BA39	+	0.18
	RIFG_BA47	→	LSFG_BA9	+	0.31
	RIFG_BA47	→	LIFG_BA47	+	0.23
	RIFG_BA47	→	LCaudate	+	0.20
Network 3					
*Inhibition*	LFFA_BA37	→	LAMY	−	−0.26
	RFFA_BA37	→	MedFG_BA10	−	−0.24
	LIOG_BA19	→	RAMY	−	−0.13
	LPHG_BA27	→	LIOG_BA19	−	−0.19
	LPHG_BA27	→	LAMY	−	−0.17
	RFFA_BA37	→	RThal	+	0.29
	RFFA_BA37	→	LAMY	+	0.20
	LAMY	→	RThal	+	0.35
	RAMY	→	LIOG_BA19	+	0.11
*Excitation*	LFFA_BA37	→	RThal	−	−0.19
	LIOG_BA19	→	MedFG_BA10	−	−0.15
	RThal	→	RFFA_BA37	−	−0.12
	RThal	→	LAMY	−	−0.24
	LPHG_BA27	→	MedFG_BA10	−	−0.38
	LPHG_BA27	→	RFFA_BA37	−	−0.19
	RAMY	→	MedFG_BA10	−	−0.30
	RAMY	→	LAMY	−	−0.31
	RThal	→	MedFG_BA10	+	0.19
	RThal	→	LFFA_BA37	+	0.14
	RThal	→	LIOG_BA19	+	0.19
	RThal	→	LPHG_BA27	+	0.14
	LAMY	→	MedFG_BA10	+	0.33
	LAMY	→	LFFA_BA37	+	0.15
	LAMY	→	RAMY	+	0.37
Network 4					
*Inhibition*	LCun_BA18	→	LINS_BA13	−	−0.19
	LINS_BA13	→	RPCC_BA30	−	−0.23
	RPCC_BA30	→	LINS_BA13	−	−0.15
	RPCC_BA30	→	RThal	−	−0.11
	RThal	→	LMOG_BA19	−	−0.14
	RPostCG_BA2	→	LPostCG_BA2	+	0.17
	RPostCG_BA2	→	LCun_BA18	+	0.11
	LSPL_BA7	→	LPostCG_BA2	+	0.36
	LSPL_BA7	→	RPCC_BA30	+	0.12
	LMOG_BA19	→	RPreCun_BA19	+	0.17
	LCun_BA18	→	RPreCun_BA19	+	0.16
	LINS_BA13	→	LCun_BA18	+	0.14
	RThal	→	LINS_BA13	+	0.27
*Excitation*	LPostCG_BA2	→	RPostCG_BA2	−	−0.28
	LPostCG_BA2	→	LCun_BA18	−	−0.22
	LPostCG_BA2	→	LINS_BA13	−	−0.27
	LPostCG_BA2	→	RThal	−	−0.14
	RPreCun_BA19	→	LMOG_BA19	−	−0.20
	RPreCun_BA19	→	RThal	−	−0.15
	LINS_BA13	→	RPostCG_BA2	−	−0.36
	RPCC_BA30	→	LPostCG_BA2	−	−0.21
	RPCC_BA30	→	LSPL_BA7	−	−0.18
	RThal	→	LSPL_BA7	−	−0.15
	RPostCG_BA2	→	LINS_BA13	+	0.28
	LMOG_BA19	→	LCun_BA18	+	0.12
	RThal	→	RPCC_BA30	+	0.25

Abbreviations: BA, Brodmann area; LAMY, left amygdala; LCaudate, left caudate; LCun, left cuneus; LFFA, left fusiform face area; LIFG, left inferior frontal gyrus; LINS, left insula; LIPL, left inferior parietal lobule; LMFG, left middle frontal gyrus; LMOG, left middle occipital gyrus; LMTG, left middle temporal gyrus; LPostCG, left postcentral gyrus; LPHG, left parahippocampal gyrus; LSFG, left superior frontal gyrus; LSPL, left superior parietal lobule; LSTG, left superior temporal gyrus; MedFG, medial frontal gyrus; RACC, right anterior cingulate cortex; RAMY, right amygdala; RFFA, right fusiform face area; RIFG, right inferior frontal gyrus; RINS, right insula; RIPL, right inferior parietal lobule; RMFG, right middle frontal gyrus; RPCC, right posterior cingulate; RPostCG, right postcentral gyrus; RPreCun, right precuneus; RThal, right thalamus.

Within N1 (fronto‐parietal), connectivity within all three brain lobes of the network (i.e., frontal, parietal, and limbic) was associated with reappraisal tendency. A reciprocal connection was identified between the frontal and parietal regions and between the frontal and limbic regions associated with reappraisal tendency. The RMFG and RPreCun emerged as the predominant regions, projecting and receiving input connections from most other ROIs in relation to reappraisal tendency. All projecting and receiving connections from the RPreCun were negatively linked to reappraisal tendency. All projecting connections from the RMFG were also negatively linked to reappraisal tendency, with input connections displaying varying associations (i.e., both positive and negative associations with reappraisal tendency). In N2 (fronto‐temporal), connections within the frontal areas were largely positively related to reappraisal tendency. Negatively associated reciprocal connections were observed between the frontal and the temporal regions, and positively associated connections between the frontal and limbic regions. The LSFG regions (BA6 and BA9) and RIFG (primarily connecting within frontal regions) were the most connected regions in association with reappraisal tendency. For N3 (temporo‐limbic), connections were observed within the limbic regions associated with reappraisal tendency, particularly between the bilateral amygdalae. Reciprocal connections were identified between temporal and limbic and between temporal and subcortical areas in relation to reappraisal tendency. The regions most associated with reappraisal tendency were the RThal and LAMY. In N4 (parieto‐limbic), connections were identified within the parietal and the occipital regions to be associated with reappraisal tendency. The LINS was the regions with the most input/output connections showing associations with reappraisal tendency, having negatively associated output and input within brain lobe connections. Input and output connections with parietal, occipital, and subcortical regions showed varying associations with reappraisal tendency.

#### 3.5.1 Prediction of Reappraisal Tendency

**FIGURE 10:**
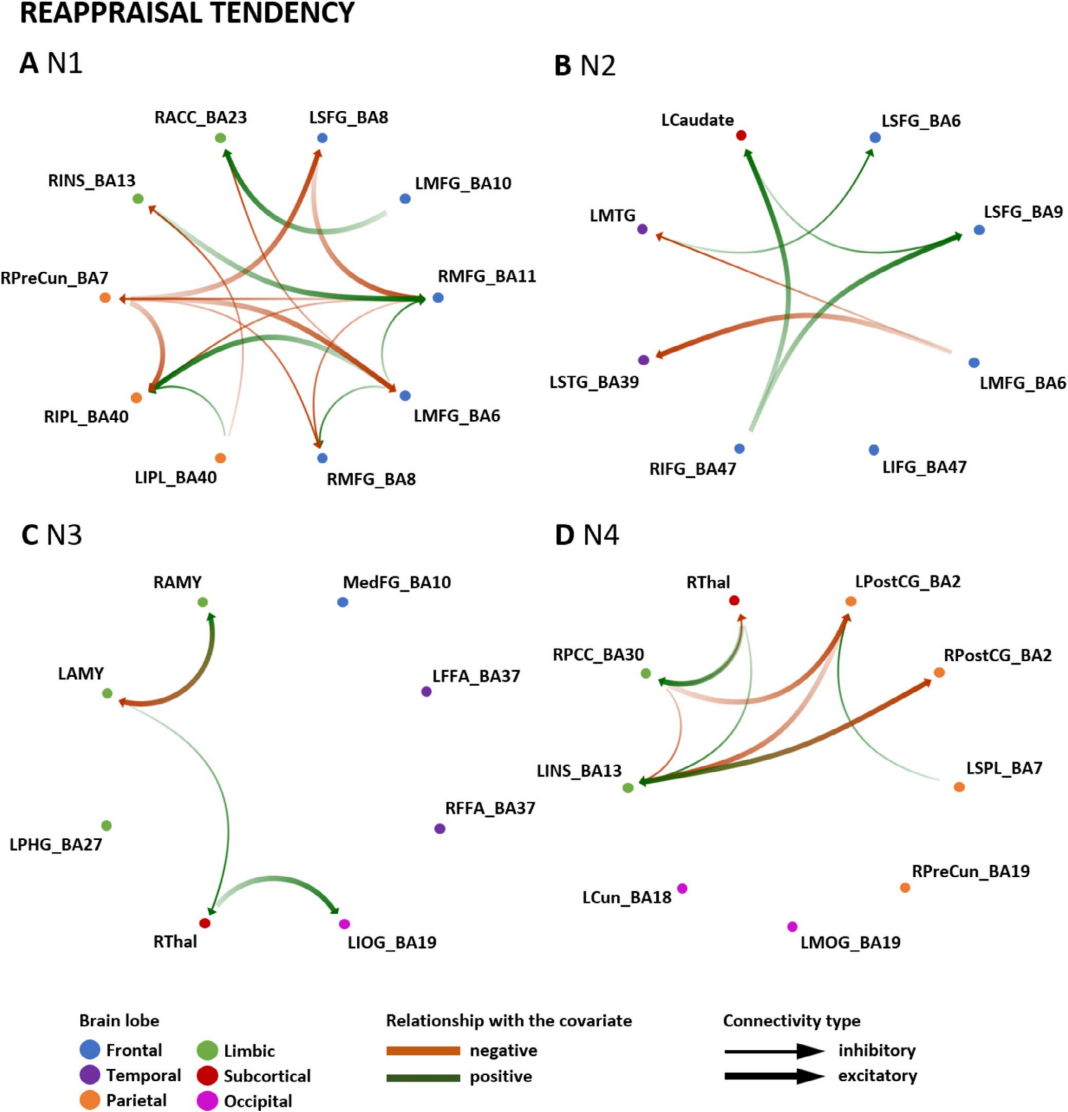
Prediction of reappraisal tendency by effective connectivity in networks N1–N4. LOOCV for the prediction of reappraisal tendency by effective connectivity in networks N1–N4 with significant effect sizes (*p* < 0.05). The green and red arrows indicate the association of distraction capacity with EC in each network (green: positive association with ER measure; red: negative association). The color saturation shows the direction of the connectivity (i.e., source to target: from less to more saturated with an arrow). The thickness of the line (i.e., thin or thick) indicates whether the connection is inhibitory or excitatory. Brain regions are organized from cortical to limbic areas. BA, Brodmann area; LAMY, left amygdala; LCaudate, left caudate; LCun, left cuneus; LFFA, left fusiform face area; LIFG, left inferior frontal gyrus; LINS, left insula; LIOG, left inferior occipital gyrus; LIPL, left inferior parietal lobule; LMFG, left middle frontal gyrus; LMOG, left middle occipital gyrus; LMTG, left middle temporal gyrus; LPHG, left parahippocampal gyrus; LPostCG, left postcentral gyrus; LSFG, left superior frontal gyrus; LSPL, left superior parietal lobule; LSTG, left superior temporal gyrus; MedFG, medial frontal gyrus; RACC, right anterior cingulate cortex; RAMY, right amygdala; RFFA, right fusiform face area; RIFG, right inferior frontal gyrus; RINS, right insula; RIPL, right inferior parietal lobule; RMFG, right middle frontal gyrus; RPCC, right posterior cingulate; RPostCG, right postcentral gyrus; RPreCun, right precuneus; RThal, right thalamus.

To test whether the observed connections could be used for a prediction of individual reappraisal tendency, we conducted a LOOCV. The results are illustrated in Figure 10 and reported in Table 5 (all connections not in bold). This analysis step aims to discern the connections specifically predictive of only reappraisal tendency. The findings demonstrated that the effect sizes observed in this LOOCV were large enough to predict an individual's reappraisal tendency with an out‐of‐sample estimate between the predicted and observed connections. In N1, 12 connections exhibited significant predictive power for reappraisal tendency. Furthermore, N2 and N3 revealed five and three connections with predictive power, respectively. Finally, 10 connections in N4 were observed that significantly contributed to the prediction of reappraisal tendency. For an overview of the correlation coefficients and associated *p*‐values for each connection, please refer to Table 5.

**TABLE 5:** Results of the leave‐one‐out cross‐validation analyses for reappraisal tendency.

Network and connection type	Connectivity			*r*‐value	*p*‐value
	Source		Target		
Network 1					
*Inhibition*	RMFG_BA11	→	RMFG_BA8	0.43	**0.00**
	RMFG_BA11	→	RIPL_BA40	0.46	0.00
	RMFG_BA11	→	RPreCun_BA7	0.30	0.03
	LMFG_BA6	→	RMFG_BA11	0.31	**0.03**
	LMFG_BA6	→	RMFG_BA8	0.30	0.03
	LMFG_BA6	→	RACC_BA23	0.31	0.03
	LIPL_BA40	→	RIPL_BA40	0.35	0.01
	LIPL_BA40	→	RINS_BA13	0.29	0.04
	RPreCun_BA7	→	RMFG_BA8	0.47	0.00
*Excitation*	LSFG_BA8	→	RMFG_BA11	0.36	**0.01**
	LMFG_BA10	→	RACC_BA23	0.27	0.04
	LMFG_BA6	→	RIPL_BA40	0.27	0.05
	RPreCun_BA7	→	LSFG_BA8	0.38	0.01
	RPreCun_BA7	→	LMFG_BA6	0.33	0.02
	RPreCun_BA7	→	RIPL_BA40	0.31	**0.02**
	RINS_BA13	→	RMFG_BA11	0.31	0.03
Network 2					
*Inhibition*	LMFG_BA6	→	LMTG	0.34	**0.02**
	LMTG	→	LSFG_BA6	0.31	0.03
	LCaudate	→	LSFG_BA9	0.27	0.05
*Excitation*	LMFG_BA6	→	LSTG_BA39	0.27	0.05
	RIFG_BA47	→	LSFG_BA9	0.30	0.03
	RIFG_BA47	→	LCaudate	0.33	0.02
Network 3					
*Inhibition*	LAMY	→	RThal	0.36	0.01
*Excitation*	RThal	→	LIOG_BA19	0.27	0.05
	LAMY	→	RAMY	0.30	0.03
	RAMY	→	LAMY	0.34	0.02
Network 4					
*Inhibition*	LSPL_BA7	→	LPostCG_BA2	0.30	0.03
	RPCC_BA30	→	LINS_BA13	0.27	0.05
	RPCC_BA30	→	RThal	0.32	0.02
	RThal	→	LINS_BA13	0.28	0.04
*Excitation*	LPostCG_BA2	→	LMOG_BA19	0.27	0.05
	LPostCG_BA2	→	LINS_BA13	0.35	0.01
	RPostCG_BA2	→	LINS_BA13	0.42	0.00
	LINS_BA13	→	RPostCG_BA2	0.27	0.04
	RPCC_BA30	→	LPostCG_BA2	0.28	0.04
	RThal	→	RPCC_BA30	0.31	0.03

*Note:* Bold values indicate overlapping connectivities between the covariates.

Abbreviations: BA, Brodmann area; LAMY, left amygdala; LCaudate, left caudate; LCun, left cuneus; LFFA, left fusiform face area; LIFG, left inferior frontal gyrus; LINS, left insula; LIPL, left inferior parietal lobule; LMFG, left middle frontal gyrus; LMOG, left middle occipital gyrus; LMTG, left middle temporal gyrus; LPostCG, left postcentral gyrus; LPHG, left parahippocampal gyrus; LSFG, left superior frontal gyrus; LSPL, left superior parietal lobule; LSTG, left superior temporal gyrus; MedFG, medial frontal gyrus; RACC, right anterior cingulate cortex; RAMY, right amygdala; RFFA, right fusiform face area; RIFG, right inferior frontal gyrus; RINS, right insula; RIPL, right inferior parietal lobule; RMFG, right middle frontal gyrus; RPCC, right posterior cingulate; RPostCG, right postcentral gyrus; RPreCun, right precuneus; RThal, right thalamus.

Most of the connections that were specifically predictive of reappraisal tendency in N1 projected from frontal regions (e.g., the bilateral MFG) to regions in the right hemisphere (e.g., RMFG (BA8), RIPL, and RACC). Another predictive connection projected from the RPreCun to the LSFG and the bilateral MFG (BA6 and BA8). Regarding specific connectivity within brain lobes, we observed one connection within frontal regions from LMFG (BA6) to RMFG (BA8) and one within parietal regions (i.e., from LIPL to RIPL). In N2, five predictive specific connections were observed. These connections projected from the RIFG to the LSFG and LCaudate, from LMFG to LSTG, and from LCaudate and LMTG to LSFG (BA6 and BA9). Furthermore, within N3, only three predictive connections could be observed. A reciprocal connection between LAMY and RAMY was observed, along with a connection from RThal to LIOG. Finally, 10 predictive connections could be identified in N4. Most of these connections were input and output connections of the bilateral postCG, projecting to and receiving input from limbic and occipital regions. Further connections were observed for RThal targeting LINS and RPCC. Notably, two reciprocal connections within N4 were predictive of reappraisal tendency: connections between the RPostCG and LINS and connections between the RThal and RPCC.

### 3.6 General ER Effects

Finally, we evaluated whether connections within the four ER networks N1–N4 were modulated by all three ER measures, that is, by distraction capacity, reappraisal capacity, and reappraisal tendency. Connections meeting this criterion are highlighted in Figure 9 (all color‐coded rectangles with dashed lines). Information on the direction of the connectivity between regions (excitatory/inhibitory) across participants can be found in Table 4.

Within N1 (fronto‐parietal), overlapping connectivity within brain lobes was identified in the frontal and parietal regions. An association with all three ER measures was observed for reciprocal connections between frontal and parietal regions and between frontal and limbic regions. The same was true for output connections of the LIPL targeting all three lobes of the network. The node with the most connections showing associations with all three ER measures was the RMFG BA11. In N2 (fronto‐temporal), only four connections showed an overlap in association with all three ER measures. These were primarily input/output connections from the LMFG. A reciprocal connection was identified between LMFG and LMTG, which was negatively associated with the three ER measures when projecting from the frontal to temporal region and positively associated with receiving input from the temporal region. Within N3 (temporo‐limbic), the only three identified overlaps in associations were observed for connections that originated from the limbic regions, that is, for connections of the LPHG projecting to occipital and frontal areas, and connections of the LAMY to the RThal. For N4 (parieto‐limbic), regarding overlap in connections within brain lobes, only one positively associated connection was observed in the parietal and one negatively associated connection within the limbic areas in relation to the three ER measures. Parietal and limbic regions had the most input/output connections showing an overlap in associations with the three ER measures. In contrast, no overlaps were observed for associations with connections involving subcortical areas. Notably, the insula had the most connections associated with the three ER measures, predominantly connecting with parietal, occipital, and other limbic regions.

#### 3.6.1 Connections Predictive of Distraction Capacity, Reappraisal Capacity, and Reappraisal Tendency

As a final step, we evaluated connections predictive of all three ER measures under study, that is, distraction capacity, reappraisal capacity, and reappraisal tendency. These connections are listed in Table 5 (all bold connections). All connections showing an overlap with regard to their predictive value for all three ER measures were located within N1 and N2. Four of these connections were observed in N1 (i.e., LSFG, LMFG (BA6), and RMFG (BA11) targeting RMFG (BA8) and RMFG (BA11), and RPreCun connecting to RIPL), and only one in N2 (from LMFG BA6 to LMTG). There were no overlaps in associations with ER capacity and tendency within N3 and N4.

## 4 Discussion

With the present study, we investigated whether and how rs‐fMRI effective connectivity within brain networks relevant for ER is associated with individual differences in ER capacity and tendency for the two strategies of reappraisal and distraction. While prior studies relating individual differences in ER to brain connectivity primarily focused on individual differences in the capacity to implement reappraisal (Morawetz et al. 2024), our findings highlight the importance of intrinsic network connectivity within key ER networks for *both* the capacity to implement as well as the tendency to select reappraisal versus distraction. Notably, our results underscore the relevance of the selection phase in the regulation process. This phase precedes implementation and has been linked to positive mental health outcomes such as well‐being and resilience (Rammensee et al. 2023). By examining effective connectivity patterns within predefined ER networks (Morawetz et al. 2020), we provide novel evidence supporting the role of large‐scale neural interactions in shaping individual differences in ER capacity and tendency. These findings contribute to a more nuanced understanding of the neural basis of ER and may inform theoretical models that differentiate between various stages of the regulation process (Gross 2015).

### 4.1 Intrinsic Neural Network Architecture Underlying Reappraisal and Distraction Capacity

Our findings reveal important commonalities in the network dynamics supporting both regulation strategies, distraction and reappraisal. Importantly, effective connectivity within N1 (fronto‐parietal) mainly correlated with ER capacity. This underscores its function as a broad cognitive control network that supports both strategies. In a similar manner, N4 was also related to overall regulatory capacity, highlighting its significance in integrating information needed for effective ER, irrespective of the particular strategy used. Interestingly, despite N2's (fronto‐temporal) established role in language processing, its involvement did not differ between reappraisal and distraction, indicating that linguistic processes are relevant for both strategies and do not explain differences in reappraisal more than distraction. Moreover, N3 (temporo‐limbic) exhibited comparable associations with capacity for both strategies, further highlighting shared regulatory mechanisms. However, within these shared networks, there were also differences in the nature of connectivity: reappraisal was predominantly associated with inhibitory connections, whereas distraction showed associations with a mix of inhibitory and excitatory connections across networks. These findings suggest that while reappraisal relies more on inhibition within regulatory networks, distraction operates through a more dynamic interplay of excitation and inhibition. These findings indicate that while distraction and reappraisal share core network dynamics that support ER more broadly, they also rely on distinct neural circuits.

Beyond the common neural mechanisms underlying distraction and reappraisal, our findings provide insight into the neural distinctions between the regulation strategies of distraction and reappraisal, highlighting differences in their dependence on network‐specific connectivity patterns and thereby extending previous findings on activity differences between these strategies (Dörfel et al. 2014). Across several networks (N1, N3, and N4), reappraisal showed more associations with intrinsic connectivity than distraction. This may be due to the fact that reappraisal is a cognitively more demanding strategy (Kanske et al. 2011; Shafir et al. 2015; Sheppes 2020), necessitating increased connectivity within these networks for effective implementation. In contrast, distraction capacity exhibited the most distinct associations with connections within N2.

Furthermore, distraction capacity showed strong associations to connectivity within N2 (fronto‐temporal), which is associated with language processing, suggesting that distraction may partially rely on inner speech or verbal redirection to shift attention away from emotional stimuli (Kalisch et al. 2006). Importantly, while both distraction and reappraisal can involve linguistic processes, their use of language differs fundamentally. Reappraisal typically engages semantic and narrative restructuring of emotional meaning—requiring deliberate reinterpretation of the stimulus—whereas distraction tends to involve a shift in attentional focus, often via verbal or cognitive content unrelated to the emotional stimulus (Kanske et al. 2011; McRae et al. 2010). Thus, the association of N2 with distraction may reflect the role of supportive verbal processes, such as subvocal rehearsal or task‐related verbal working memory, rather than the meaning‐making central to reappraisal. N1 demonstrated associations with both ER strategies, highlighting its domain‐general role in cognitive control. This supports the notion that N1 facilitates general preparatory processes—such as maintaining goals or task sets—regardless of the specific regulatory tactic employed (Schweizer et al. 2013). Distraction capacity was characterized by widespread associations to connectivity involving the prefrontal regions of N1 and N2 and the parietal components of N4. The presence of associations with both inhibitory and excitatory connections for these regions suggests a role for the dynamic coordination of attention, where prefrontal regions support top‐down modulation and parietal areas contribute to attentional shifting or maintenance, aligning with previous findings of frontoparietal recruitment in distraction (Dörfel et al. 2014; Kanske et al. 2011; McRae et al. 2010). This pattern reflects the inter‐network integration needed for the successful implementation of distraction. Nonetheless, these connections were not related to distraction capacity in a consistent manner, as we observed both positive and negative associations, suggesting variable individual strategies or compensatory mechanisms. In contrast, reappraisal capacity was characterized by extensive associations with prefrontal and parietal connectivity within N1, along with distinct contributions from N2, N3, and N4. This broader set of associations supports the idea that reappraisal requires more integrative, meaning‐based processing, involving semantic restructuring and internal monitoring, which recruit a wider array of functional systems (Morawetz, Bode, et al. 2016; Picó‐Pérez et al. 2017; Steward et al. 2021).

Although reappraisal was associated with broader and more integrated network connectivity across N1–N4, our findings indicate that not all of these connections consistently relate to individual differences in reappraisal capacity. This suggests that both strategies may be supported by widespread connectivity within and across networks, but only specific connections vary in a way that explains between‐person differences (Rammensee et al. 2023). In this sense, the overall connectivity pattern reflects the general architecture underlying ER, yet individual variability in ER capacity appears to depend on a selective subset of connections, particularly within prefrontal and parietal regions (Kanske et al. 2015; Li, Yang, et al. 2021; Strauss et al. 2016). Thus, functional networks provide a common substrate for ER, but individual differences in connectivity are key to understanding who is more or less successful in strategy implementation (Rammensee et al. 2023).

Our findings highlight the crucial role of N1 (frontoparietal) and N4 (parieto‐limbic) in predicting reappraisal capacity, with both networks showing the highest predictive power in the LOOCV. Similarly, numerous connections within N1 and N4 also contributed to the prediction of distraction capacity, underscoring their shared role in supporting effective ER. However, a key insight from our prediction analysis is that the most predictive connections were not those broadly involved in both strategies, but the distinct, non‐overlapping connections specific to each strategy. This suggests that ER capacity is best predicted by the unique network configurations supporting either reappraisal or distraction, rather than by connections generally involved in regulatory mechanisms. In other words, while N1 and N4 are important across both strategies, their effectiveness in predicting capacity is driven by the specific, strategy‐dependent connectivity patterns rather than their general involvement in ER. These findings reinforce the idea that successful ER relies on strategy‐specific neural adaptations rather than shared regulatory networks (Dörfel et al. 2014; Kanske et al. 2011; Liu et al. 2022; Vrtička et al. 2011).

### 4.2 Intrinsic Neural Network Architecture Underlying Reappraisal Tendency

The observed associations between ER measures and effective connectivity also provide insight into the neural mechanisms underlying the tendency to select reappraisal over distraction. A key finding is the high number of associations with connectivity within frontal and parietal cortices of N1 and within frontal regions of N2 and parietal regions of N4. These associations suggest that widespread connectivity across cognitive control networks is associated with reappraisal tendency. Notably, the distinct connections in N2 and N3 that predicted reappraisal tendency did not overlap with those predicting reappraisal capacity, indicating that different network dynamics contribute to the selection and implementation of reappraisal. In N1 and N2, inhibitory and excitatory connections were equally associated with reappraisal tendency, with no clear predominance of direction in association, highlighting the complexity of the underlying neural processes. While N3 and N4 showed a slight predominance of excitatory connections, their associations with reappraisal tendency remained mixed.

Notably, the association between reappraisal tendency and amygdala‐to‐medial frontal influences showed opposite signs across hemispheres. Such divergence is compatible with known hemispheric nuances of amygdala function and the functional heterogeneity of the medial frontal cortex. In prevailing models, reappraisal engages prefrontal control systems that interact with—and typically modulate—amygdala responses, while vmPFC/pgACC contributes valuation, safety, and extinction‐related processes. Accordingly, stronger bottom‐up amygdala signaling may relate to different—and even opposing—associations with reappraisal preference depending on the specific medial–frontal subnetworks involved (Baas et al. 2004; Berboth and Morawetz 2021; Buhle et al. 2014; Morawetz, Bode, Baudewig, and Heekeren 2017). Taken together, these results suggest that while multiple brain networks contribute to the tendency to use reappraisal, the interaction between bottom‐up emotional processing and top‐down control plays a critical role in shaping individual differences in strategy selection (McRae et al. 2012; Ochsner et al. 2009; Otto et al. 2014; Wang and Yin 2023).

### 4.3 Shared Intrinsic Neural Network Architecture Underlying Regulation Capacity and Tendency

The observed commonalities in the associations of effective connectivity with distraction capacity, reappraisal capacity, and reappraisal tendency point to shared underlying regulatory mechanisms. At the same time, the comparison between ER capacity and tendency also illustrates key distinctions in how these processes are implemented at the neural level. Within N1, the associations with reciprocal connections between frontal and parietal regions and between frontal and limbic areas suggest a central role for these regions in supporting both the capacity and the tendency to engage in ER. The prominence of the right middle frontal gyrus as the node with the most connections, showing associations with all three ER measures, may reflect its involvement in integrating cognitive and affective signals necessary for both selecting and successfully implementing regulation strategies. Similarly, the left inferior parietal lobule appears to function as a key hub, facilitating interaction across different regulatory networks. These connectivity patterns align with the idea that ER is not driven by isolated regions but rather by coordinated networks.

Despite the shared role of N1 (frontoparietal), the connections in N2 (frontotemporal) that showed overlapping associations were notably sparse, not exceeding the number of four connections that were primarily located within the frontal lobe. This suggests that while frontal regions contribute to both ER tendency and capacity, they do so more modularly, possibly reflecting different neural computations for selection and implementation (Etkin et al. 2015). The observed reciprocal connections between the left middle frontal gyrus and the left middle temporal gyrus, with opposite associations to ER capacity and tendency depending on input vs. output, are particularly intriguing. This bidirectional relationship suggests a dynamic interplay between executive and semantic processes, potentially indicating that individual differences in linguistic or conceptual elaboration during ER contribute to strategy selection and implementation differences.

The limited overlap in N3 (temporo‐limbic) and N4 (parieto‐limbic) further underscores the notion that while certain regions contribute to both ER tendency and capacity, others are more specialized. The limbic‐driven projections observed in N3, particularly from the parahippocampal gyrus and the amygdala, indicate that emotional salience processing may be similarly engaged across all ER measures but in a way that does not necessarily predict individual differences in strategy use. The centrality of the insula in N4 and its association with all ER measures, with its widespread connections to parietal, occipital, and limbic regions, reinforces its well‐established role in interoceptive and emotional integration (Stern et al. 2017). However, the fact that no predictive overlapping connections were found in N3 and N4 suggests that while these networks contribute to the processing of emotional salience and interoception, they may not play a consistent role in explaining individual differences in ER capacity or tendency (Morawetz and Basten 2024).

Taken together, these findings suggest that while there are core networks that support both the capacity to regulate emotions as well as the tendency to prefer specific strategies, the most predictive connections tend to be those that are distinct rather than shared (Liu et al. 2022; Otto et al. 2014). This highlights the importance of strategy‐specific neural adaptations, where the capacity and tendency for a given regulation strategy are shaped by dedicated connectivity patterns rather than a single, uniform regulatory network (Dörfel et al. 2014; Fine et al. 2022). These insights contribute to a more nuanced understanding of the neural architecture of ER, emphasizing the interplay between shared connectivity supporting cognitive control mechanisms and more specialized, strategy‐dependent connectivity.

### 4.4 Limitations

While our study provides novel insights into the neural mechanisms underlying ER capacity and tendency, several limitations must be acknowledged.

First, to keep our analyses relatively compact and accessible, we focused exclusively on high‐intensity emotional stimuli, which may not fully capture the variability in ER responses across different levels of emotional arousal (Morawetz et al. 2024; Silvers, Weber, et al. 2015). Future studies should examine whether our findings generalize to low‐ or moderate‐intensity stimuli, as regulatory demands may differ depending on the emotional strength of the stimulus.

Second, our analysis was constrained to predefined networks and within‐network connectivity, without considering cross‐network interactions. Although this approach allowed us to investigate network‐specific contributions to ER capacity and tendency, effective ER likely depends on dynamic interactions between networks. Future studies should explore cross‐network connectivity patterns to provide a more comprehensive understanding of the neural architecture of ER.

Third, because DCM parameters exhibit partial collinearity, multivariate predictors that include all edges require large samples, strict nested cross‐validation, and ideally external validation to yield reliable biomarkers. Our goal in the present study was hypothesis‐driven mapping of specific *directed* pathways rather than maximal prediction. Future work should evaluate whether regularized multivariate approaches (e.g., elastic net, PLS/PLSR) provide incremental out‐of‐sample gains over single‐edge models, assessed with permutation testing and independent test sets.

Fourth, our study focused on resting‐state rs‐fMRI effective connectivity rather than task‐based connectivity. Because rs‐fMRI is measured outside the regulation context and our behavioral measures were acquired at a different time point, our conclusions concern trait‐like baseline coupling that may scaffold regulation, rather than the state‐dependent mechanisms engaged during reappraisal or distraction. This interpretive stance reduces the risk of reverse inference but necessarily limits causal claims. Prior work indicates that resting‐state connectivity shapes task‐evoked activation and predicts performance (Cole et al. 2016, 2021; Smith et al. 2009; Tavor et al. 2016), and observed similarities between task‐based and resting‐state functional connectivity suggest that resting architecture may reflect pathways through which cognitive processes operate. However, the direct relationship between resting‐state effective connectivity and task‐related network dynamics during emotion regulation remains insufficiently characterized. A stringent next step—aligned with arguments by Finn (2021) and planned for a separate study—would be a within‐subject comparison of resting‐state and emotion‐regulation task‐based effective connectivity (e.g., spectral DCM or related approaches) during reappraisal/distraction or passive viewing, to test how context shifts coupling patterns and whether resting architecture explains variance in task‐evoked dynamics. Design features such as acquiring rest before the task or introducing a washout period would help mitigate carryover effects. Given the transdiagnostic relevance of ER capacity (Aldao et al. 2016; Cludius et al. 2020; Kring and Sloan 2010; Morawetz et al. 2025; Sloan et al. 2017), identifying reliable biological markers remains a critical goal (Horwitz and Rowe 2011). Our findings represent a step toward this aim while underscoring the need for the task‐based comparisons outlined above.

Of note, resting‐state effective connectivity may be influenced by factors only indirectly related to ER—such as vigilance/arousal, fatigue, physiological noise, head motion, and hormonal status. While we implemented standard quality control (no participant exceeded 3 mm displacement; models showed high variance explained and low posterior parameter correlations), we acknowledge these as limitations to the interpretability of the associations observed for rs‐fMRI. Critically, we could not directly compare resting‐state and ER task‐based effective connectivity because we did not collect fMRI during the ER tasks. Future studies should perform head‐to‐head comparisons to delineate how trait‐like intrinsic coupling relates to state‐evoked regulatory dynamics and how both are related to individual differences in emotion regulation capacity and tendency.

Furthermore, while split‐half reliability for the ER tendency index was moderate and comparable to earlier estimates, we did not obtain multi‐session data to assess test–retest stability over longer intervals. Future work should evaluate temporal stability (days/weeks) to further substantiate trait‐level inferences.

Sample characteristics also limit our findings, as we only investigated healthy young adults. This limits the generalizability of our results with regard to clinical populations, who often show altered ER and network connectivity patterns. Future studies should include larger and more diverse samples, particularly individuals with psychopathology, to assess whether resting‐state effective connectivity can serve as a diagnostic tool for ER deficits. Several clinical groups, including those with borderline personality disorder, substance use disorder, depression, and anxiety disorders, have been reported to exhibit impairments in ER (Aldao et al. 2010; Eftekhari et al. 2009; Gross and Jazaieri 2014; Taylor and Liberzon 2007; Werner and Gross 2010). Additionally, ER develops significantly across the lifespan, with adolescence being a critical period marked by structural and functional changes, particularly in prefrontal‐amygdala connectivity. Some studies suggest this period is associated with a decrease in amygdala reactivity, interpreted as a sign of improved emotional control and regulatory maturation (Silvers et al. 2012, 2016b, 2017; Silvers, Shu, et al. 2015). Interpreting connectivity and activation patterns without accounting for developmental variability may oversimplify the complexity of ER mechanisms during this transitional period. Investigating resting‐state effective connectivity in developmental and clinical populations may provide insight into early markers of ER difficulties, which could be targeted through intervention strategies such as ER training (Denny 2018; Denny et al. 2015; Denny and Ochsner 2014).

Finally, our sample (*N* = 40) and resting‐state scan (~10 min) are at the lower bound for brain–behavior association studies, which constrains power, widens confidence intervals, and may inflate effect‐size estimates. To quantify this, a sensitivity analysis of our second‐level models (controlling for age and sex; df = 36) shows that, at α = 0.05 with 80% power, the minimum detectable association is approximately partial *r* ≈0.43 (Δ*R* ^2^ ≈0.19; Cohen's *f* ^2^ ≈0.23). Detecting more moderate effects would require substantially larger samples (*N* ≈86 for *r* = 0.30; *N* ≈124 for *r* = 0.25). Large‐scale analyses indicate that reproducible brain‐wide association studies (BWAS) typically require substantially larger cohorts (often in the thousands), and recent work quantifies how prediction accuracy increases with both sample size and total scan time per participant—with sample size ultimately exerting the larger influence. Practical recommendations from this work suggest aiming for ≥ 30 min scan time when feasible. Our findings should therefore be viewed as preliminary mechanistic evidence that requires independent replication in larger samples and, ideally, with longer scans. In line with these recommendations, future studies will prioritize increased N and extended rs‐fMRI duration, alongside pre‐registered analyses and external validation (Marek et al. 2022; Ooi et al. 2025; Tervo‐Clemmens et al. 2023).

By addressing these limitations, future research can refine our understanding of how resting‐state network dynamics contribute to ER and explore their potential as biomarkers for individual differences in ER and psychopathology.

## 5 Conclusion

Our findings provide evidence that all examined networks show effective connectivity related to ER capacity and the tendency to select reappraisal over distraction in the face of high‐intensity emotional stimuli. However, the most pronounced associations were observed for the connectivity of frontal regions in network N1, highlighting this network as fundamental for strategy selection and implementation. The substantial predictive value of N1 underscores its role as a core regulatory network, facilitating both general and strategy‐specific preparatory processes.

Notably, in N1, both inhibitory and excitatory connections showed positive and negative associations with regulation capacity and tendency, indicating no clear pattern of direction in the associations. This complex interplay within frontal regions suggests a high degree of functional flexibility, potentially allowing the system to adapt to situational demands and explaining individual differences in regulatory preferences. Such flexibility may be crucial for effective ER in dynamic real‐world environments, where the selection and implementation of strategies must be tailored to changing emotional and cognitive contexts (Li et al. 2024; Pruessner, Barnow, and Holt 2020; Pruessner, Barnow, Holt, Joormann, and Schulze 2020; Specker et al. 2024; Ullah et al. 2018).

Beyond the general role of N1 across strategies, we also identified strategy‐specific associations with effective connectivity in other networks. These findings indicate that some degree of specificity in regulation processes can already be observed at the level of resting‐state connectivity, representing a preparatory neural state that influences the selection and implementation of a particular strategy. Importantly, these changes in connectivity were predictive of behavioral outcomes, reinforcing the notion that resting‐state network dynamics serve as a crucial foundation for individual differences in ER.

In sum, our results suggest that while ER involves both general and strategy‐specific neural mechanisms, resting‐state connectivity plays a key role in shaping regulatory capacity and tendency (Li, Xie, et al. 2021; Morawetz et al. 2024; Picó‐Pérez et al. 2018; Wang et al. 2024; Zanella et al. 2022). Future research should explore how these intrinsic network configurations interact with task‐activated regulation processes and how they may serve as biomarkers for individual differences in ER and psychopathology (Yamashita et al. 2024).

## Supporting information


**Data S1:** Supporting Information.

## Data Availability

The data that support the findings of this study are available from the corresponding author upon reasonable request.
