# Supplementary material for: Wired to Regulate: Brain Connectivity Predicts Emotion Regulation Capacity and Tendency
Source: Hum Brain Mapp. 2025 Nov 8;46(16):e70400. doi: 10.1002/hbm.70400 (PMC12595545; doi:10.1002/hbm.70400)
Supplement: Supplementary file 1 — Data S1: Supporting Information. [file HBM-46-e70400-s001.docx]

**SUPPLEMENTAL INFORMATION**

**Wired to Regulate: Brain Connectivity Predicts Emotion Regulation Capacity and Tendency**

C. Morawetz^1^_,_ M. Hajrić^1^, R. A. Rammensee^2^, S. Berboth^3^, U. Basten^2^

^1^ Department of Psychology, University of Innsbruck, Austria

^2^ Department of Psychology, RPTU Kaiserslautern-Landau, Germany

^3^ Department of Neurology, Charité Universitätsmedizin Berlin, Germany


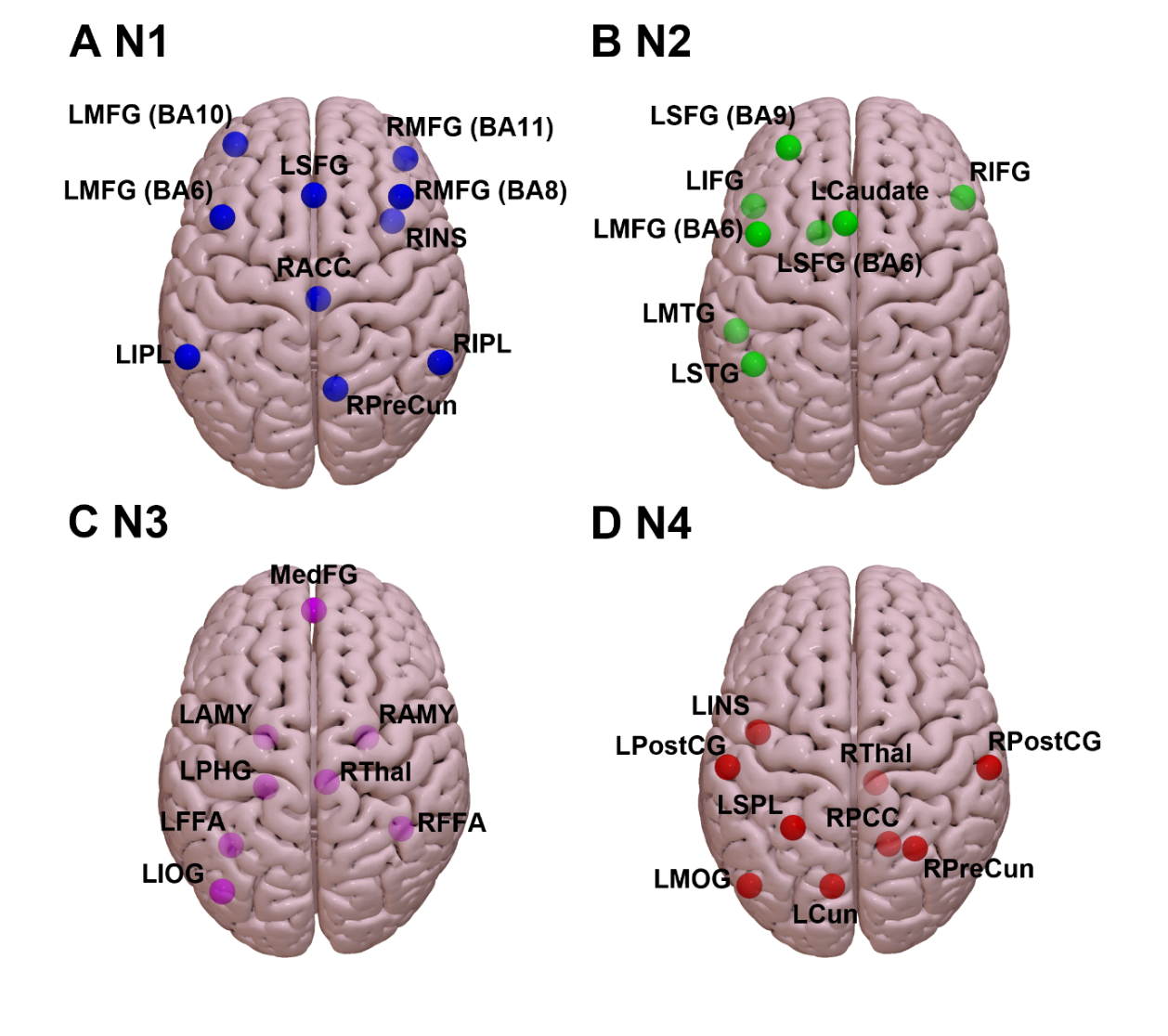


**Supplemental Figure S1**. Overview of the ROI locations within the four predefined networks. A: N1 - frontal (5), parietal (3), and limbic (2) regions. B: N2 - frontal (5), temporal (2), and subcortical (1) regions. C: N3 - frontal (1), temporal (2), occipital (1), subcortical (1), and limbic (3) regions. D: N4 - parietal (4), occipital (2), limbic (2), and subcortical (1) regions. The intensity of the ROI color indicates how close to the surface of the cortex it is located (i.e., more saturated colors indicate cortical regions and less saturated subcortical and limbic regions). BA, Brodmann area; LAMY, left amygdala; LCaudate, left caudate; LCun, left cuneus; LFFA, left fusiform face area; LIFG, left inferior frontal gyrus; LINS, left insula; LIOG, left inferior occipital gyrus; LIPL, left inferior parietal lobule; LMFG, left middle frontal gyrus; LMOG, left middle occipital gyrus; LMTG, left middle temporal gyrus; LPHG, left parahippocampal gyrus; LPostCG, left postcentral gyrus; LSFG, left superior frontal gyrus; LSPL, left superior parietal lobule; LSTG, left superior temporal gyrus; MedFG, medial frontal gyrus; RACC, right anterior cingulate cortex; RAMY, right amygdala; RFFA, right fusiform face area; RIFG, right inferior frontal gyrus; RINS, right insula; RIPL, right inferior parietal lobule; RMFG, right middle frontal gyrus; RPCC, right posterior cingulate; RPostCG, right postcentral gyrus; RPreCun, right precuneus; RThal, right thalamus. Figure created in Surf Ice software (<http://www.nitrc.org/projects/surfice/>).

| **Supplemental Table S1**. MNI coordinates of the four predefined neural networks (N1-N4) implicated in ER (Morawetz et al., 2020). | | | | | | | |
| --- | --- | --- | --- | --- | --- | --- | --- |
|  |  |  |  |  | Coordinates | | |
| Networks | Side | Region | BA | Volume | x | Y | z |
| Network 1 | L | Superior Frontal Gyrus | 8 | 11704 | 0 | 24 | 50 |
|  | R | Middle Frontal Gyrus | 8 | 11024 | 40 | 24 | 42 |
|  | R | Inferior Parietal Lobule | 40 | 9968 | 58 | -52 | 38 |
|  | L | Inferior Parietal Lobule | 40 | 6216 | -58 | -50 | 44 |
|  | L | Middle Frontal Gyrus | 10 | 4664 | -36 | 52 | -2 |
|  | L | Middle Frontal Gyrus | 6 | 4288 | -42 | 14 | 48 |
|  | R | Middle Frontal Gyrus | 11 | 2792 | 42 | 46 | -8 |
|  | R | Insula | 13 | 2000 | 36 | 16 | 6 |
|  | R | Cingulate Gyrus | 23 | 1336 | 2 | -22 | 30 |
|  | R | Precuneus | 7 | 944 | 10 | -64 | 36 |
| Network 2 | L | Inferior Frontal Gyrus | 47 | 19464 | -46 | 24 | -8 |
|  | L | Superior Frontal Gyrus | 6 | 16592 | -4 | 10 | 62 |
|  | R | Inferior Frontal Gyrus | 47 | 6856 | 50 | 28 | -8 |
|  | L | Superior Temporal Gyrus | 39 | 6704 | -46 | -52 | 28 |
|  | L | Middle Temporal Gyrus | * | 5024 | -54 | -34 | -2 |
|  | L | Middle Frontal Gyrus | 6 | 4568 | -44 | 6 | 50 |
|  | L | Superior Frontal Gyrus | 9 | 3080 | -30 | 48 | 26 |
|  | L | Caudate | * | 1960 | -16 | 10 | 12 |
| Network 3 | L | Amygdala | * | 8640 | -22 | -4 | -16 |
|  | R | Amygdala | * | 6512 | 24 | -4 | -18 |
|  | R | Fusiform Gyrus | 37 | 4776 | 40 | -46 | -18 |
|  | R | Thalamus | * | 3528 | 6 | -26 | 0 |
|  | L | Fusiform Gyrus | 37 | 1256 | -38 | -54 | -14 |
|  | L | Parahippocampal Gyrus | 27 | 1216 | -22 | -28 | -4 |
|  | B | Medial Frontal Gyrus | 10 | 1016 | 0 | 54 | -10 |
|  | L | Inferior Occipital Gyrus | 19 | 912 | -42 | -76 | -6 |
| Network 4 | L | Postcentral Gyrus | 2 | 4160 | -58 | -22 | 32 |
|  | L | Insula | 13 | 3752 | -44 | -4 | 10 |
|  | L | Superior Parietal Lobule | 7 | 2240 | -28 | -52 | 56 |
|  | R | Postcentral Gyrus | 2 | 1736 | 62 | -22 | 30 |
|  | L | Cuneus | 18 | 1224 | -10 | -76 | 22 |
|  | L | Middle Occipital Gyrus | 19 | 1152 | -48 | -74 | 2 |
|  | R | Thalamus | * | 1024 | 10 | -26 | -4 |
|  | R | Precuneus | 19 | 832 | 28 | -60 | 38 |
|  | R | Posterior Cingulate | 30 | 832 | 16 | -56 | 16 |
| Side: L=left, R=right, B=bilateral. BA, Brodmann area. | | | | | | | |


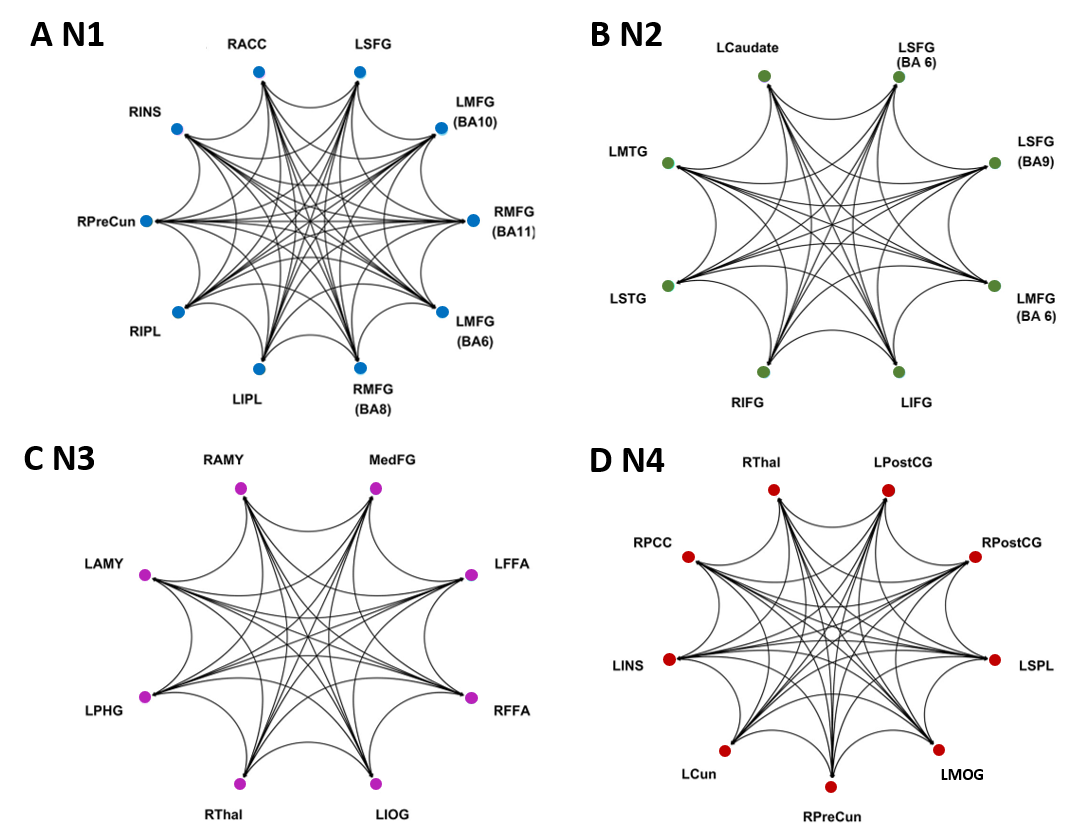


**Supplemental Figure S2**. Fully connected models of all four networks (N1-N4) and all participants. N1: 10 nodes, 100 connections. N2: 8 nodes, 64 connections. N3: 8 nodes, 64 connections. N4: 9 nodes, 81 connections. BA, Brodmann area; LAMY, left amygdala; LCaudate, left caudate; LCun, left cuneus; LFFA, left fusiform face area; LIFG, left inferior frontal gyrus; LINS, left insula; LIOG, left inferior occipital gyrus; LIPL, left inferior parietal lobule; LMFG, left middle frontal gyrus; LMOG, left middle occipital gyrus; LMTG, left middle temporal gyrus; LPHG, left parahippocampal gyrus; LPostCG, left postcentral gyrus; LSFG, left superior frontal gyrus; LSPL, left superior parietal lobule; LSTG, left superior temporal gyrus; MedFG, medial frontal gyrus; RACC, right anterior cingulate cortex; RAMY, right amygdala; RFFA, right fusiform face area; RIFG, right inferior frontal gyrus; RINS, right insula; RIPL, right inferior parietal lobule; RMFG, right middle frontal gyrus; RPCC, right posterior cingulate; RPostCG, right postcentral gyrus; RPreCun, right precuneus; RThal, right thalamus.


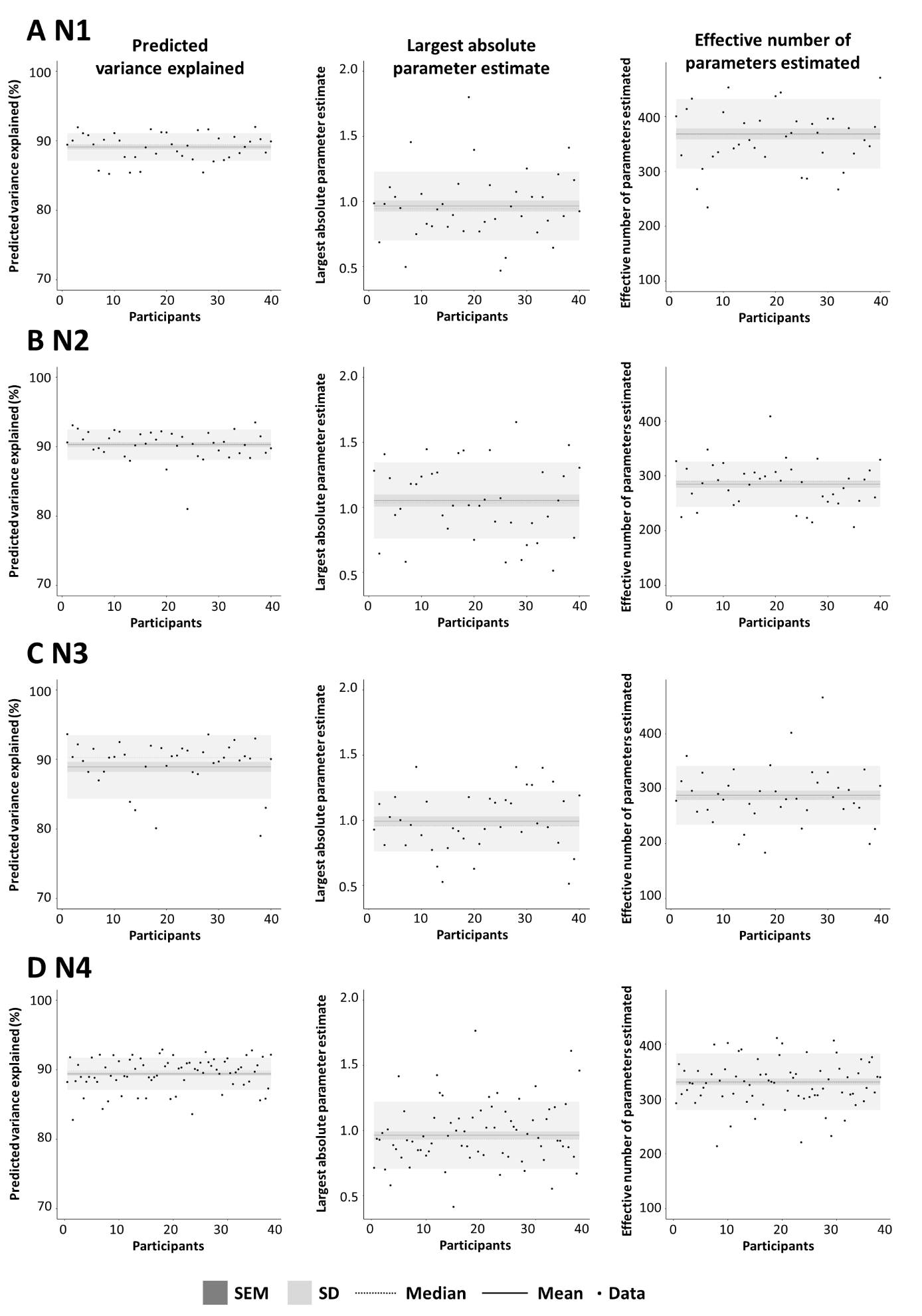


**Supplemental Figure S3**. Model convergence statistics indicating good model convergence. Left column: Predicted variance explained for all participants was above the minimum threshold of 10%, suggesting that the estimated models adequately capture a significant portion of the observed variance in the data. Middle column: Largest absolute parameter estimate was above the typical connection strength of 1/8 Hz, suggesting that the estimated connections in the model have meaningful magnitudes and are not weak. Third column: The effective number of parameters estimated in terms of divergence between posterior and prior parameter densities, suggesting that the estimated parameters are stable and not overly influenced by noise or sampling variability.


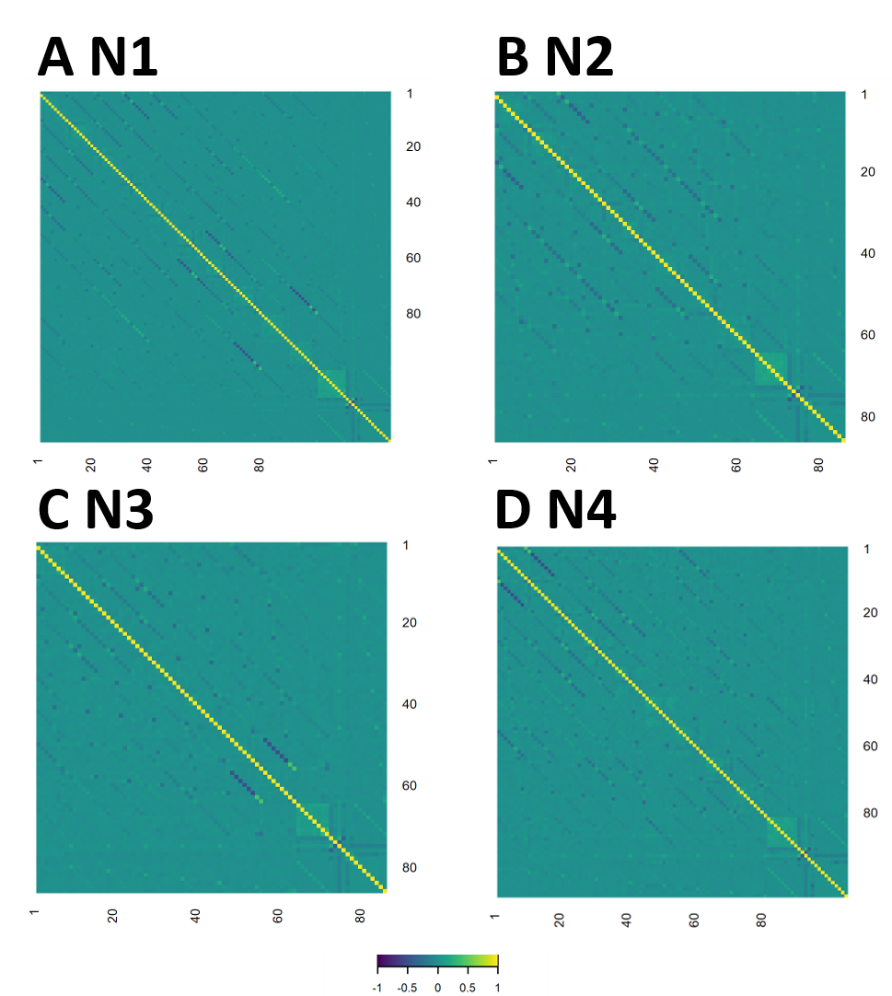


**Supplemental Figure S4**. Low posterior correlations among all parameters indicating identifiable parameters, suggesting that the estimated connectivity strengths and modulatory effects are reliable and can be interpreted with confidence. Low (i.e., close to zero) values suggest that the parameters are relatively independent and provide unique information about the connectivity patterns, whereas high correlation values (i.e., close to -1 and 1) indicate redundancies or collinearity between parameters. Diagnostic statistics were obtained via spm_dcm_fmri_check (Zeidman et al., 2019).
